# Overcoming effector T cell exhaustion in ovarian cancer ascites with a novel adenovirus encoding for a MUC1 bispecific antibody engager and IL-2 cytokine

**DOI:** 10.1016/j.ymthe.2024.07.009

**Published:** 2024-07-19

**Authors:** Saru Basnet, Mirte Van der Heijden, Dafne C.A. Quixabeira, Elise Jirovec, Susanna A.M. Grönberg-Vähä-Koskela, James H.A. Clubb, Anna Kanerva, Santeri Pakola, Lyna Haybout, Victor Arias, Otto Hemminki, Tatiana Kudling, Sadia Zafar, Victor Cervera-Carrascon, Joao M. Santos, Akseli Hemminki

## Main text

(Molecular Therapy, *32*, https://doi.org/10.1016/j.ymthe.2024.06.029; September 2024)

In the originally published version of this article, Victor Cervera-Carrascon was mistakenly omitted from the author list. Dr. Cervera-Carrascon along with other authors designed the experiments for the study, and him being missing from the author list was the result of an error by the authors who assembled the author list. The authors apologize for any confusion this may have caused, and this error has been corrected in the paper online.

