# Supplementary material for: Overcoming effector T cell exhaustion in ovarian cancer ascites with a novel adenovirus encoding for a MUC1 bispecific antibody engager and IL-2 cytokine
Source: Mol Ther. 2024 Jun 22;32(9):3114–27. doi: 10.1016/j.ymthe.2024.06.029 (PMC11403222; doi:10.1016/j.ymthe.2024.06.029)
Supplement: Document S1. Figures S1‒S15 and Table S1 [file mmc1.pdf]

YMTHE, Volume *32*

Supplemental Information

Overcoming effector T cell exhaustion in ovarian cancer ascites with a novel adenovirus encoding for a MUC1 bispecific antibody engager and IL-2 cytokine

Saru Basnet, Mirte Van der Heijden, Dafne C.A. Quixabeira, Elise Jirovec, Susanna A.M. Grönberg-Vähä-Koskela, James H.A. Clubb, Anna Kanerva, Santeri Pakola, Lyna Haybout, Victor Arias, Otto Hemminki, Tatiana Kudling, Sadia Zafar, Victor Cervera-Carrascon, Joao M. Santos, and Akseli Hemminki

Table S1. List of antibodies used in this study.

Experiment	Antibodies	Manufacturer
T cell activation assay (in vitro) and immunological analysis of tumor (in vivo)	FITC anti-human CD3 (clone OKT3)	Biolegend, California, USA
	AF-700 anti- human CD4 (clone A161A1)	
	BV-421 anti- human CD8 (clone SK1)	
	PE-Cy™7 anti- human CD69 (clone FN50)	
	PE-Cy™5 anti- human CD25 (clone M-A251)	
	PE-CF594 anti-human CD127 (clone HIL-7R-M21)	BD Sciences, New Jersey, USA
	APC anti-human Tbet (clone 4B10)	Biolegend, California, USA
	PE anti-human EOMES (clone X4-83)	BD Sciences, New Jersey, USA
Expression of cell surface MUC1 antigen	APC anti-human MUC1 (clone 16A)	Biolegend, California, USA
Profiling of NK and NK-T like cells	BV-605 anti-human CD56 (HCD56)	Biolegend, California, USA
Activation of γδ T cell	PE anti-human TCR-γδ (clone 11F2)	Miltenyi, Bergisch Gladbach, Germany
	FITC anti-human Vδ1 (clone REA173)	
	APC anti-human Vδ2 (clone REA771)	
	AF-700 Perforin (clone B-D48)	
	BV421 GranzymeB (clone GB11)	BD Sciences, New Jersey, USA
	BV650 Interferon gamma (clone 4S.B3)	Biolegend, California, USA
T cell exhaustion panel	FITC anti-human CD3 (clone OKT3)	BioLegend, California, USA
	AF-700 anti-human CD4 (clone A161A1)	
	BV-510 anti-human CD8 (clone SK1)	BD Sciences, New Jersey, USA
	PE anti-human TIM-3 (clone A18087E)	BioLegend, California, USA
	BV-605 anti-human TIGIT (clone A15153G)	
	PE anti-human TIM-3 (clone A18087E)	
	BV-605 anti-human TIGIT (clone A15153G)	
	PE-Cy™5 anti-human LAG-3 (clone 11C3C65)	
	PE-Cy7 anti-human BTLA (clone MIH26)	
	APC anti-human CTLA-4 (clone BNI3)	
	PE-CF594 anti-human PD-1 (clone NAT105)	
	PE anti-human TOX (clone TXRX10)	Invitrogen, Massachusetts, USA
	BV-421 anti-human TCF-1 (clone S33-966)	BD Sciences, New Jersey, USA

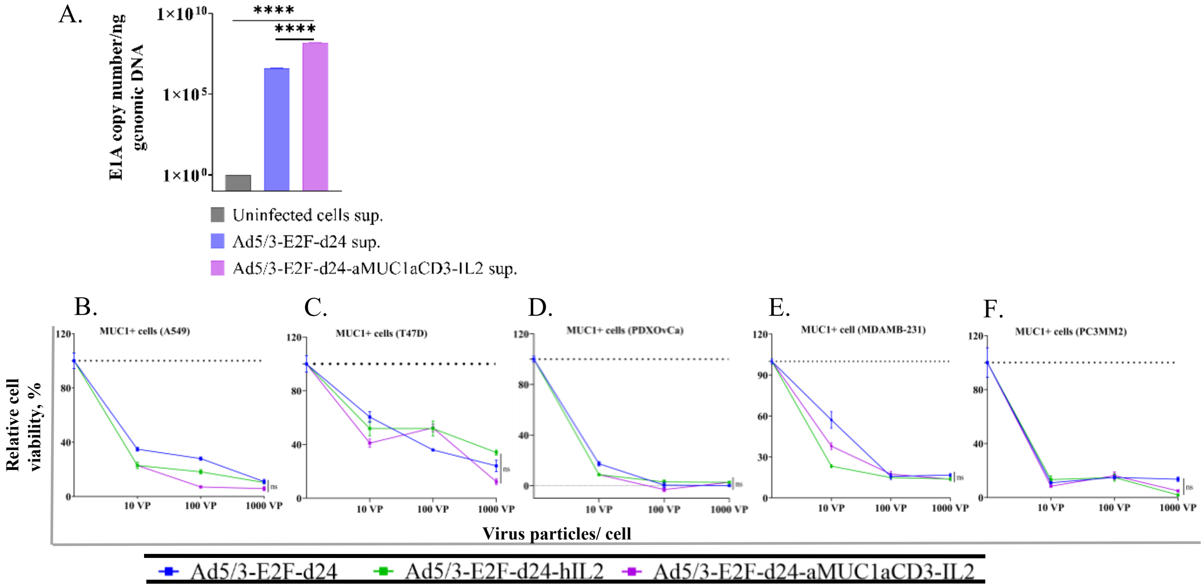

Figure S1. Oncolytic activity of TILT-322 *in vitro*. (A) Quantification of E1A gene copy number as a measure of virus replication in TILT-322 infected A549 cells supernatant. **(B-F)** Relative cell viability of human cancer cells followed by the infection of Ad5/3-E2F-d24, Ad5/3-E2F-d24-hIL2, and TILT-322 virus different concentration of virus ranging from 10, 100 or 1000 VP/ cell. Cells were incubated with the viruses for 3 days (A549, PDX-OvCa, and MDAMB-231), 5 days (PC3MM2), or 7 days (T47D) before determining cell viability. The untreated control group is indicated by the dashed line.

All experiments were run in quadruplet, and the resulting data are presented as mean ± SEM. One-way ANOVA with Tukey's post hoc test was used to compare more than two groups in the activation assay. Statistical significance is represented as *p < 0.05, ***p < 0.001, ****p < 0.0001 and ns = non-significant.

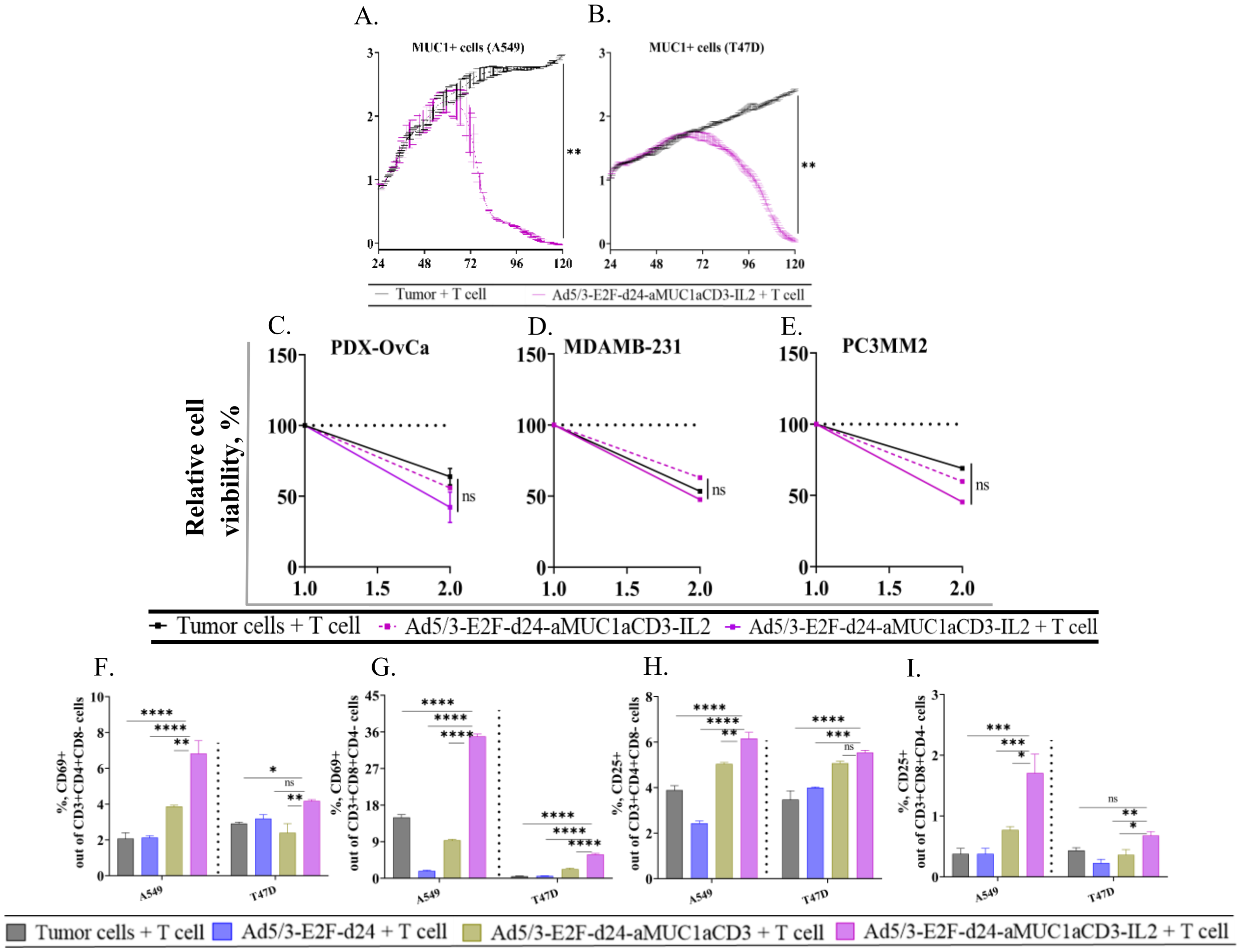
A.
MUC1+ cells (A549)
B.
MUC1+ cells (T47D)
Tumor + T cell
Ad5/3-E2F-d24-aMUC1aCD3-IL2 + T cell
C.
PDX-OvCa
D.
MDAMB-231
E.
PC3MM2
Relative cell viability, %
Tumor cells + T cell
Ad5/3-E2F-d24-aMUC1aCD3-IL2
Ad5/3-E2F-d24-aMUC1aCD3-IL2 + T cell
F.
%, CD69+ out of CD3+CD4+CD8- cells
G.
%, CD69+ out of CD3+CD8+CD4- cells
H.
%, CD25+ out of CD3+CD4+CD8- cells
I.
%, CD25+ out of CD3+CD8+CD4- cells
A549
T47D
Tumor cells + T cell
Ad5/3-E2F-d24 + T cell
Ad5/3-E2F-d24-aMUC1aCD3 + T cell
Ad5/3-E2F-d24-aMUC1aCD3-IL2 + T cell

Figure S2. **TILT-322 mediated tumor cell killing and T cell activation *in vitro*.** Real-time iCELLigence-based cytotoxicity assay showing the killing of MUC1-positive **(A)** A549 and **(B)** T47D tumor cells in the presence of virus and T cells at E:T ratio of 1 for up to 120 hours. **(C-E)** Evident of faster tumor cell killing by TILT-322 in the presence of unstimulated human CD3+ cells at E:T ratio of 5 and 1 pfu/cell virus concentration 48 hours post-infection. **(F-G)** Percentage of activated CD69+ and **(H-I)** CD25+ lymphocytes in TILT-322 treated tumors. All experiments were run in quadruplet, and the resulting data are presented as mean ± SEM. Welch's t-test was used to determine statistical significance in the iCELLigence assay and one-way ANOVA with Tukey's post hoc test was used to compare more than two groups in the activation assay. Statistical significance is represented as *p < 0.05, ***p < 0.001, ****p < 0.0001 and ns = non-significant. Statistical significance in xCELLigence assay is represented as *p = 0.0282, and **p = 0.0057.

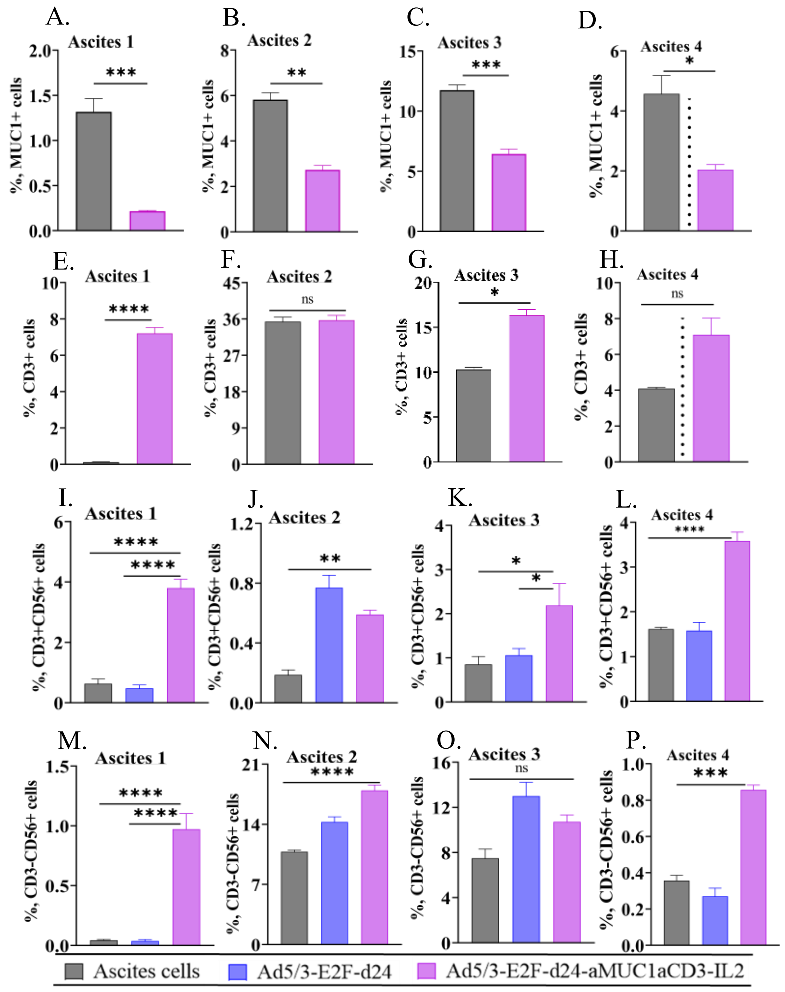

Figure S3. Detection of MUC1, T cells, and other cells followed by TILT-322 infection in ascites samples. (A-D) Detection of reduced proportion of MUC1 antigen in ascites cells and **(E-H)** Total percentage of CD3+ T cell detected in ascites samples **(I-L)** Total percentage of CD3+CD56+ cells and **(M-P)** CD3-CD56+ NK cells followed detected in four ascites samples infected with TILT-322 virus. The resulting data are presented as mean ± SEM of quadruplet. One-way ANOVA with Tukey’s post hoc test was used to run the statistical significance and the result is represented as *p < 0.05, **p < 0.01, ***p < 0.001, and ****p < 0.0001.

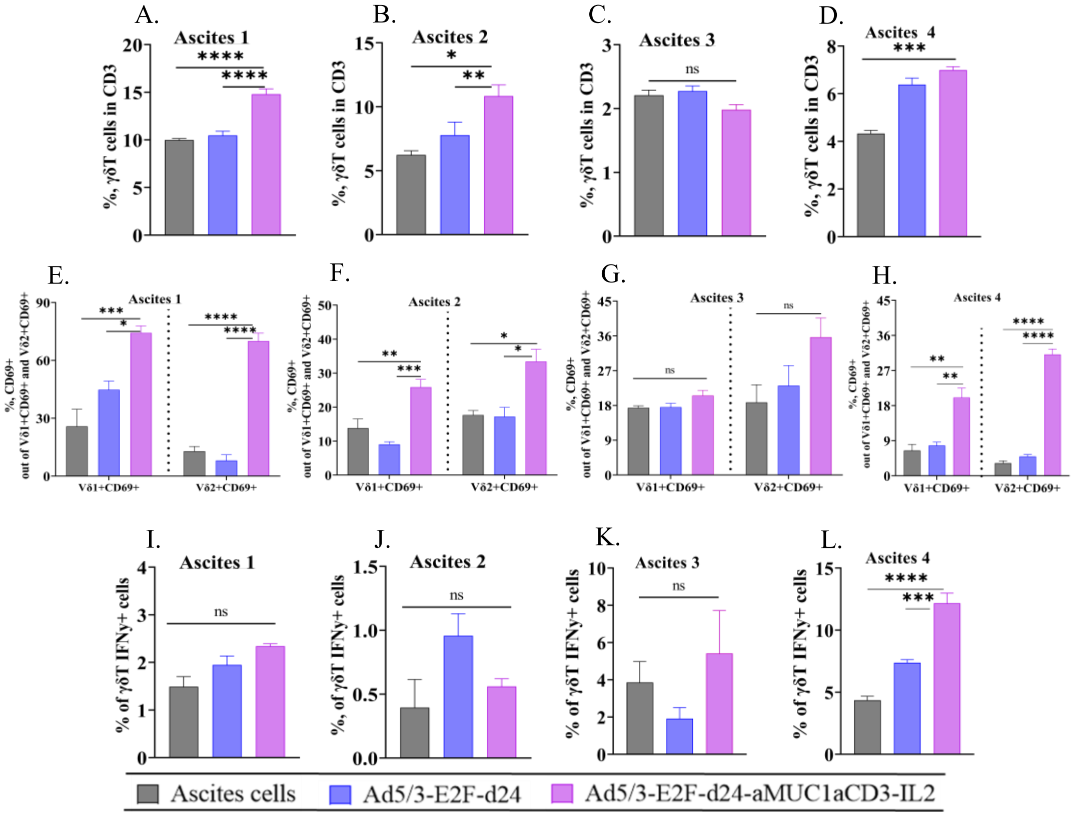

Figure S4. TILT-322 mediated activation of γδT cell.

The graph shows the percentage of **(A-D)** γδ T cells and **(E-H)** Percentage of activated CD69+ cells among the Vδ1+ and Vδ2+ subsets. **(I-L)** Interferon-gamma positive γδ T cells in all three tested ascites samples infected by TILT-322. The resulting data are presented as mean ± SEM of quadruplet. One-way ANOVA with Tukey's post hoc test was used to run the statistical significance is represented as ***p < 0.001, ****p < 0.0001 and, ns = non-significant.

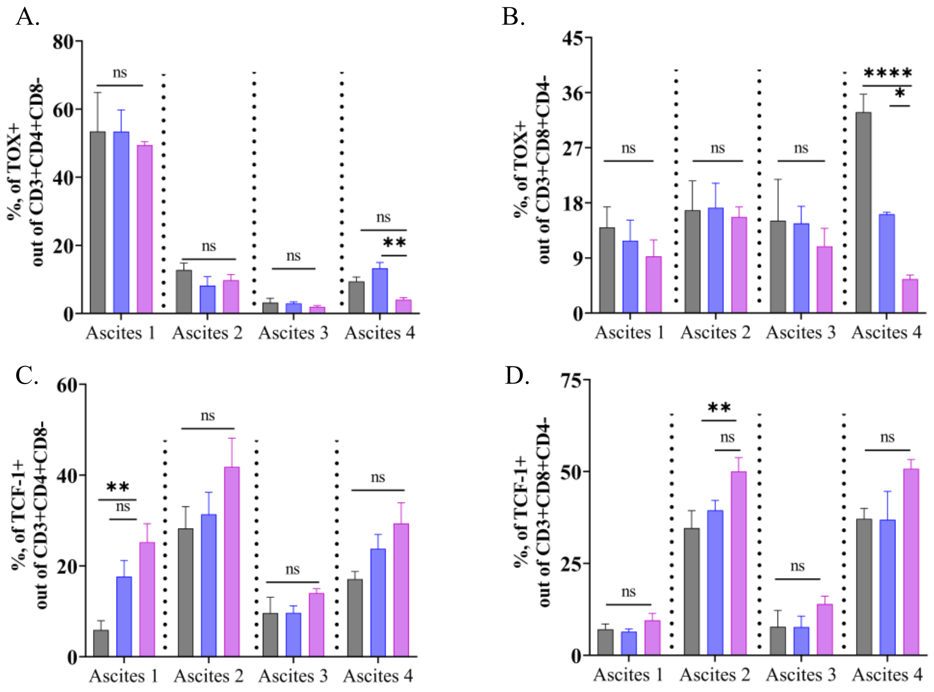

Figure S5. T cell exhaustion at the transcriptional level followed by the TILT-322 treatment. The graph shows the percentage of **(A-B)** TOX+ cells and **(C-D)** TCF-1+ cells on CD4+ and CD8+ T cells followed by TILT-322 treatment in four different ascites samples. The resulting data are presented as mean ± SEM of quadruplet. One-way ANOVA with Tukey's post hoc test was used to run the statistical significance is represented as ***$p < 0.001$, ****$p < 0.0001$ and, ns = non-significant.

A.

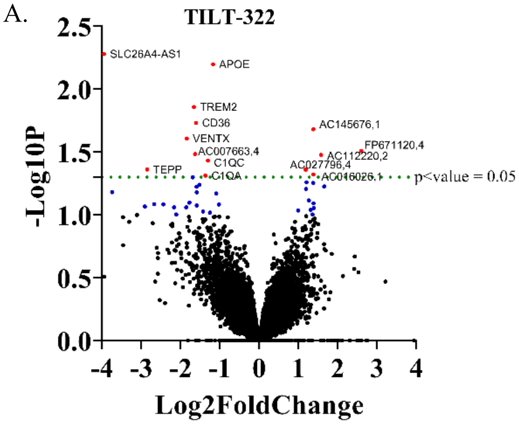

Figure S6. Differentially expressed genes in TILT-322 infected ascites cells. TILT-322 infection of ovarian ascites samples led to downregulation of 14 and upregulation of 5 differentially expressed genes in ascites samples.

A.

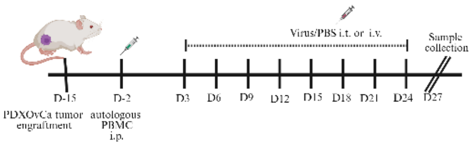

Group	Dose	Route
PBMC	1 x 10^7	Intraperitoneal
Ad5/3-E2F-d24	1 x 10^9	Intratumoral
Ad5/3-E2F-d24 aMUC1aCD3	1 x 10^9	Intratumoral
Ad5/3-E2F-d24 aMUC1aCD3-IL2	1 x 10^9	Intratumoral
Ad5/3-E2F-d24 aMUC1aCD3-IL2	1 x 10^{10}	Intravenous

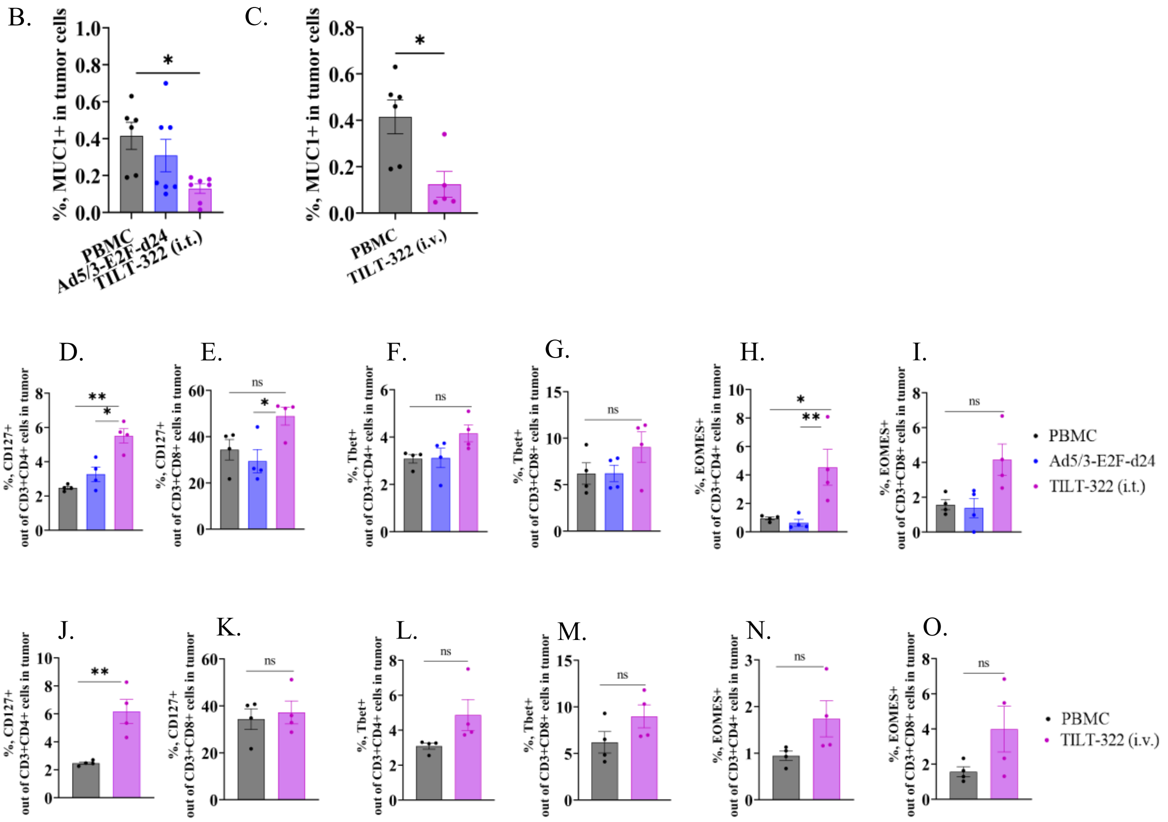

Figure S7. Schedule for the treatment of TILT-322 *in vivo* experiment and immunological analysis of animal tumor samples. (A) Schematic representation of TILT-322 animal experiment with dosage information. **(B)** Total percentage of MUC-1 positive cells detected in TILT-322 virus-treated tumors with intratumoral and **(C)** intravenous delivery. Total percentage of **(D-E)** CD127+ cells, **(F-G)** T-bet+ cells, **(H-I)** EOMES+ cells on CD4+ or CD8+ T cells in *in vivo* tumor samples followed by the local delivery of TILT-322. When the TILT-322 was given systemically, the total percentage of (**J-K)** CD127+ cells, **(L-M)** T-bet+ cells, **(N-O)** EOMES+ cells on CD4+ or CD8+ T cells in *in vivo* tumor samples is shown. The resulting data are presented as mean ± SEM of quadruplet. One-way ANOVA with Tukey's post hoc test was used to run the statistical significance is represented as ***p < 0.001, ****p < 0.0001, and, ns = non-significant.

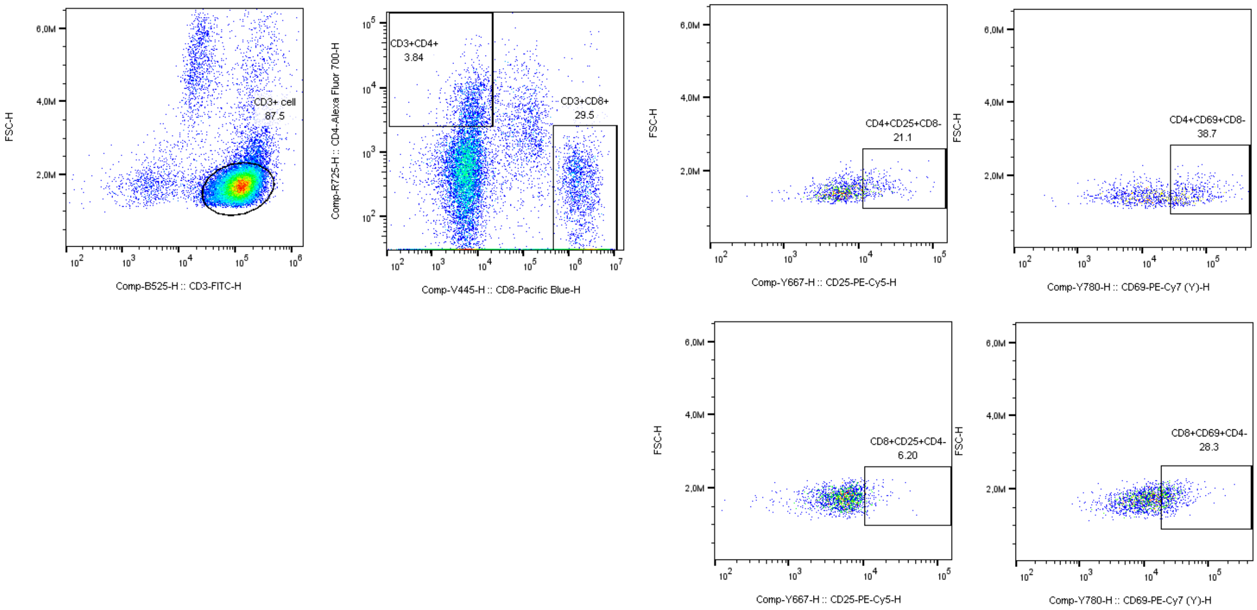

Figure S8. Flow gating strategy used to access activated CD69 and CD25 T cells *in vitro*. Gating is done from lymphocytes → CD3 → CD4 → CD69+/CD25+ or CD3 → CD8 → CD69+/CD25+.

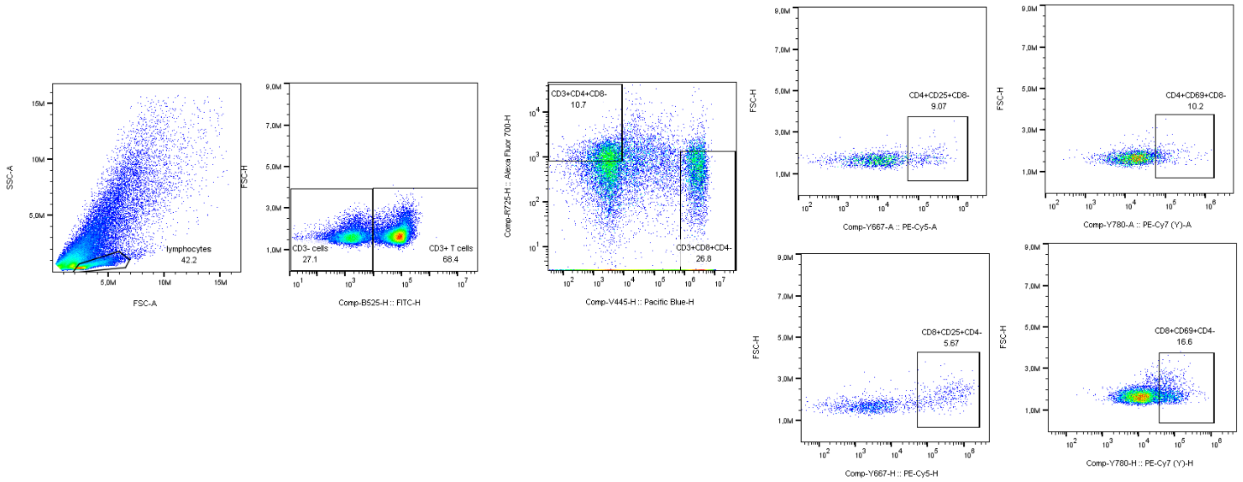

Figure S9. Flow gating strategy used to access activated CD69 and CD25 T cell *ex vivo*. Gating is done from lymphocytes→CD3+→CD4+→CD69+/CD25+ or →CD3+→CD8+→CD69+/CD25+.

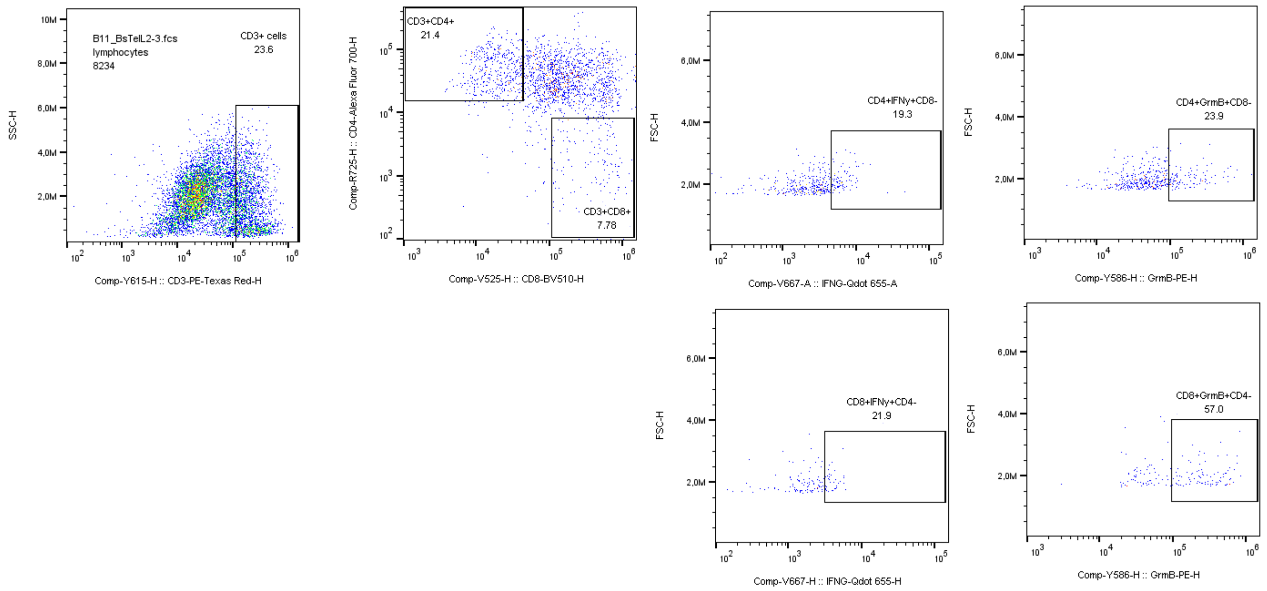

Figure S10. Flow Gating Strategy used to access granzymeB and interferon-gamma positive cells in TILT-322 infected ascites samples. Gating is done from lymphocytes→CD3+→CD4+→GranzymeB+/Interferon-y+ or →CD3+→CD8+→ GranzymeB+/Interferon-y+.

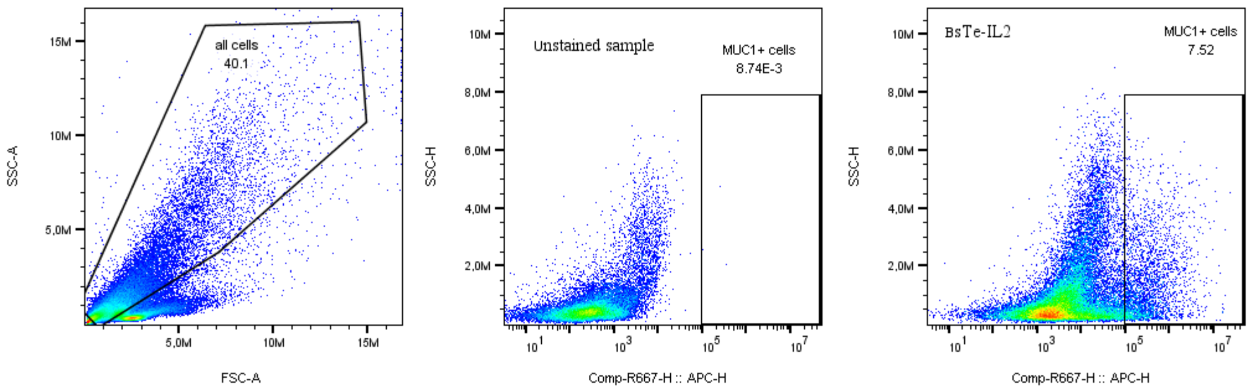

Figure S11. Flow Gating Strategy used to access MUC1+ cells out of all ascites cells. Gating is done from all cells → MUC1. MUC1 Fluorescence Minus One Control and unstained sample control were used to set the gating thresholds.

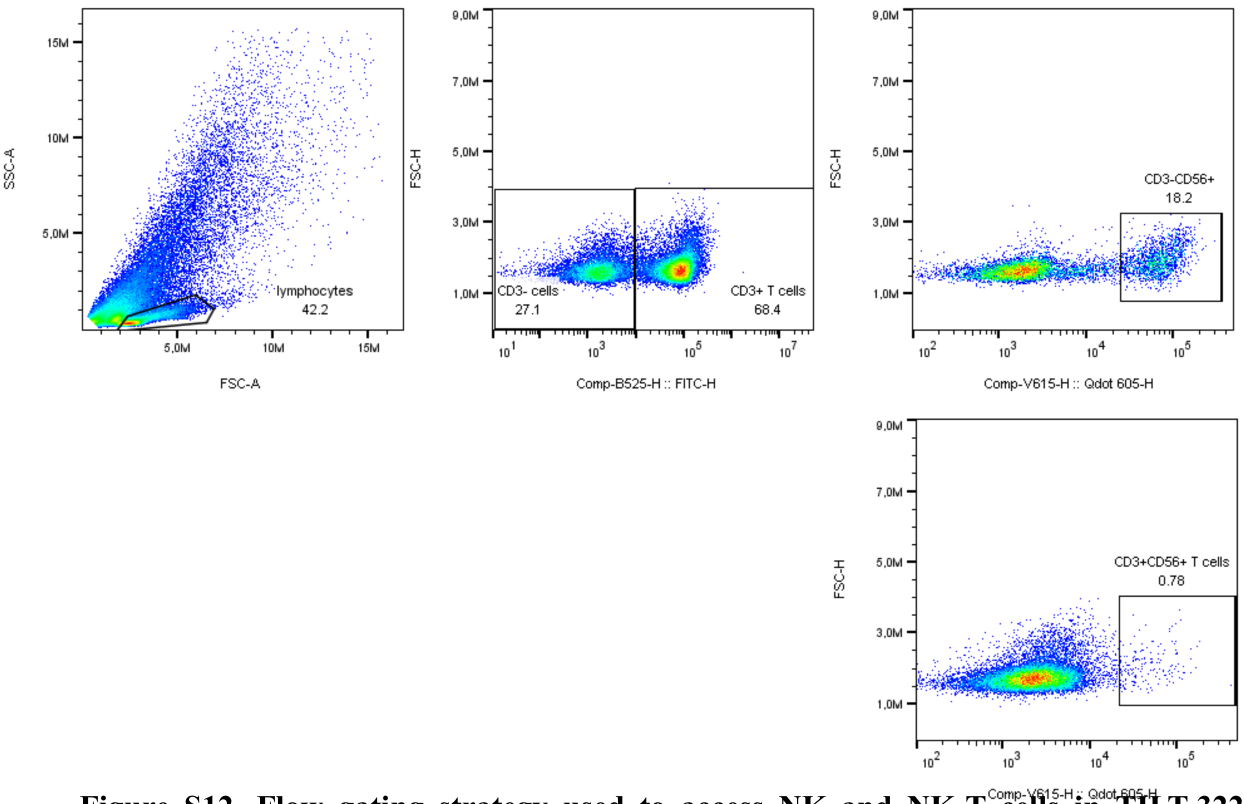

Figure S12. Flow gating strategy used to access NK and NK-T cells in TILT-322 infected ascites samples. Gating is done from lymphocytes → CD3+ cells → CD3+CD56+ cells or lymphocytes→ CD3- cells → CD3-CD56+ cells. All required Fluorescence Minus One Control and unstained sample control was used to set the gating thresholds.

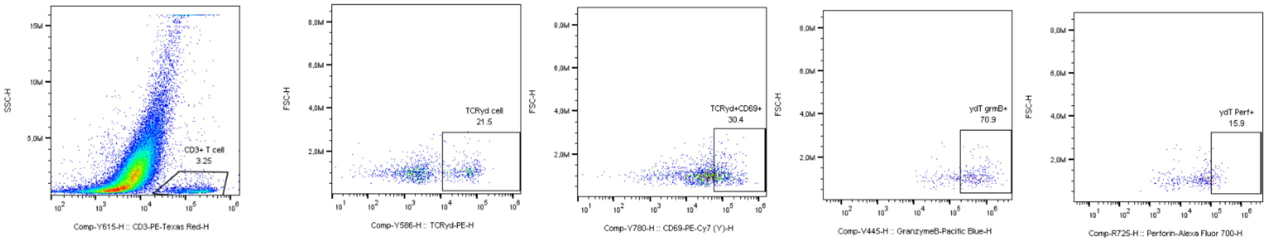

Figure S13. Flow gating strategy used to access TCR gamma delta T cell activation in TILT-322 treated ascites samples *ex vivo*. Gating is done from lymphocytes → CD3+ cells → TCRγd+ cells → CD69+ cells/ granzymeB+/ Perforin+ cells. All required Fluorescence Minus One Control and unstained sample control was used to set the gating thresholds.

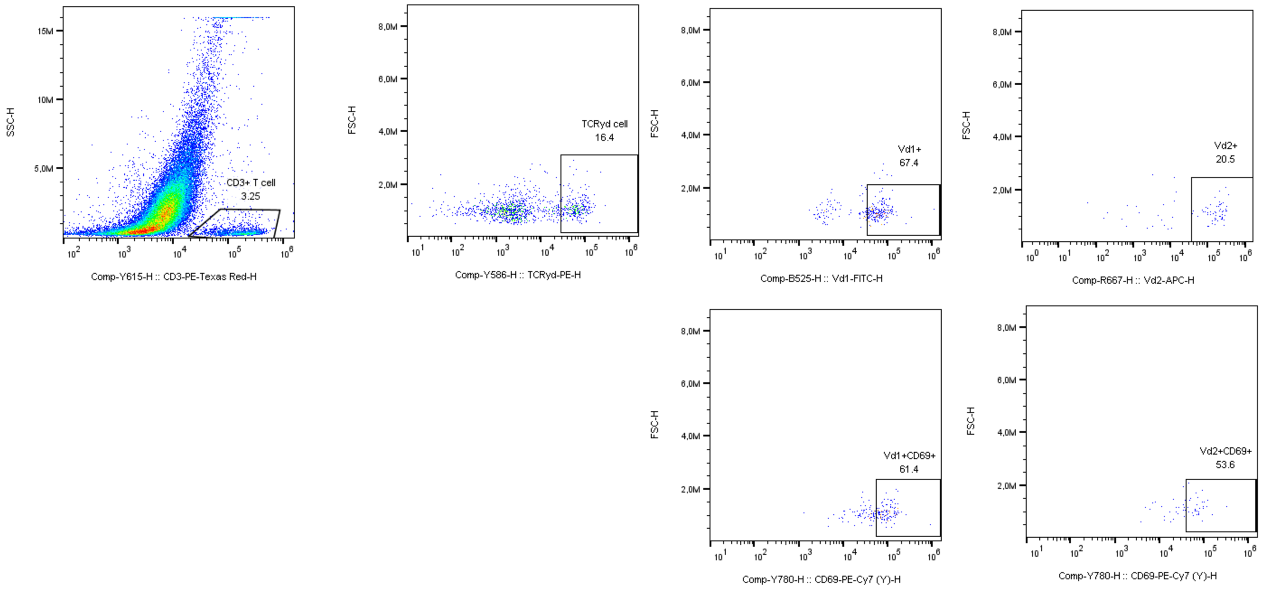

Figure S14. Flow gating strategy used to access Vd1/Vd2 gamma delta T cell subset activation. Gating is done from lymphocytes → CD3+ cells → TCRyd+ cells → Vd1+cells → CD69+ or lymphocytes → CD3+ cells → TCRyd+ cells → Vd2+cells → CD69+ cells. All required Fluorescence Minus One Control and unstained sample control was used to set the gating thresholds.

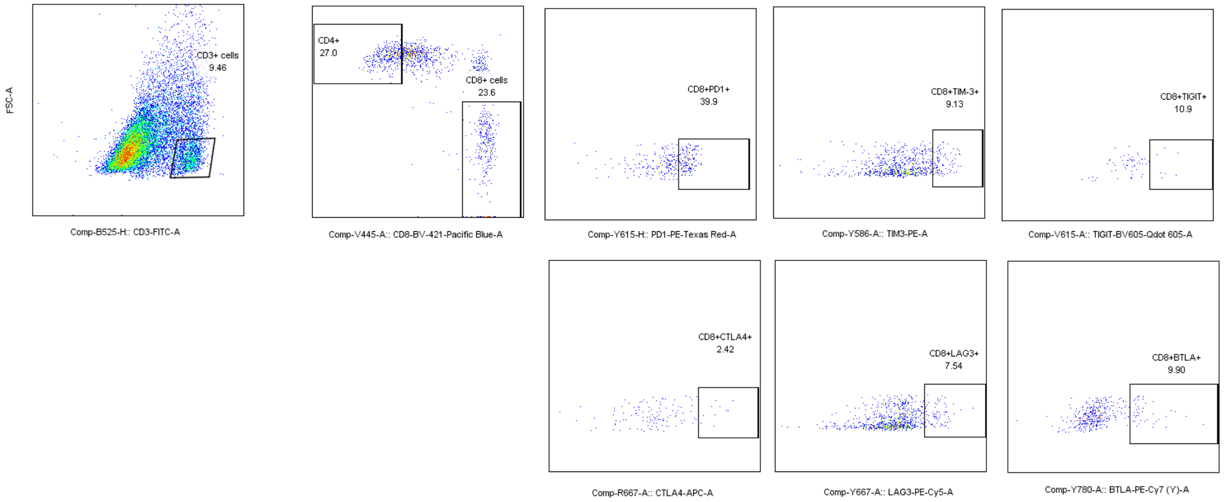

Figure S15. Flow gating strategy used to examine T cell exhaustion in ovarian ascites followed by the TILT-322 treatment. Gating is done from CD3+ cells → CD4+ → BTLA/CTLA-4/LAG-3/PD-1/TIGIT/TIM-3. A similar gating is done to access the expression on CD8+ T cells (CD3+ cells → CD8+ → BTLA/CTLA-4/LAG-3/PD-1/TIGIT/TIM-3). All required Fluorescence Minus One Control and unstained sample control was used to set the gating thresholds.
