# Supplementary material for: Overcoming effector T cell exhaustion in ovarian cancer ascites with a novel adenovirus encoding for a MUC1 bispecific antibody engager and IL-2 cytokine
Source: Mol Ther. 2024 Jun 22;32(9):3114–27. doi: 10.1016/j.ymthe.2024.06.029 (PMC11403222; doi:10.1016/j.ymthe.2024.06.029)
Supplement: Document S2. Article plus supplemental information [file mmc2.pdf]

Molecular Therapy
Original Article

# Overcoming effector T cell exhaustion in ovarian cancer ascites with a novel adenovirus encoding for a MUC1 bispecific antibody engager and IL-2 cytokine

Saru Basnet,[1,3] Mirte Van der Heijden,[1,3] Dafne C.A. Quixabeira,[1,2,3] Elise Jirovec,[1,3] Susanna A.M. Grönberg-Vähä-Koskela,[1,4] James H.A. Clubb,[1,2,3] Anna Kanerva,[1,5] Santeri Pakola,[1,3,4] Lyna Haybout,[1,2,3] Victor Arias,[1,3] Otto Hemminki,[1,6] Tatiana Kudling,[1,3] Sadia Zafar,[7,8] Victor Cervera-Carrascon,[1,2] Joao M. Santos,[1,2] and Akseli Hemminki[1,2,3,4]

[1]Cancer Gene Therapy Group, Translational Immunology Research Program, Faculty of Medicine, University of Helsinki, Helsinki, Finland; [2]TILT Biotherapeutics Ltd, Helsinki, Finland; [3]Translational Immunology Research Program (TRIMM), Research Program Unit (RPU), University of Helsinki, Helsinki, Finland; [4]Helsinki University Hospital (HUS), Comprehensive Cancer Center, Helsinki, Finland; [5]Department of Gynecology and Obstetrics, Helsinki University Hospital, Helsinki, Finland; [6]Department of Urology, Helsinki University Hospital, Helsinki, Finland; [7]Applied Tumor Genomics HUS Comprehensive Cancer Center, Research Program, Research Program Unit, University of Helsinki, Helsinki, Finland; [8]Department of Pathology, HUSLAB, HUS Diagnostic Center, University of Helsinki and Helsinki University Hospital, Helsinki, Finland

**T cell-focused cancer immunotherapy including checkpoint inhibitors and cell therapies has been rapidly evolving over the past decade. Nevertheless, there remains a major unmet medical need in oncology generally and immuno-oncology specifically. We have constructed an oncolytic adenovirus, Ad5/3-E2F-d24-aMUC1aCD3-IL-2 (TILT-322), which is armed with a human aMUC1aCD3 T cell engager and IL-2. TILT-322 treatment stimulated T cell cytotoxicity through the increased presence of granzyme B, perforin, and interferon-gamma. Additional immune profiling indicated TILT-322 increased gamma delta T cell activation and impacted other cell types such as natural killer cells and natural killer-like T cells that are traditionally involved in cancer immunotherapy. TILT-322 treatment also decreased the proportion of exhausted CD8$^{+}$ T cells as demarked by immune checkpoint expression in ovarian ascites samples. Overall, our data showed that TILT-322 treatment led to an enhanced T cell activation and reversed T cell exhaustion translating into high antitumor efficacy when given locally or intravenously. The analysis of blood and tumors isolated from an *in vivo* patient-derived ovarian cancer xenograft model suggested TILT-322 mediated tumor control through improved T cell functions. Therefore, TILT-322 is a promising novel anti-tumor agent for clinical translation.**

## INTRODUCTION

Oncolytic viruses are an effective immunotherapy with the remarkable ability to trigger antitumor T cell responses, effectively transforming immune-deprived cold tumors into inflamed hot tumors, recognizable by the immune system.[1,2] The mechanism of action of oncolytic immunotherapy involves three key elements: direct lytic cancer cell death, dispersion of tumor epitopes to induce *de novo* immune responses, and the production of immunogenic danger signals that recruit effector immune cells.[2] Arming oncolytic adenoviruses with T cell engagers and other immunostimulatory molecules holds the potential to augment these effects. In this translational study, we constructed and characterized a new oncolytic adenovirus TILT-322 (Ad5/3-E2F-d24-aMUC1aCD3-IL-2) in clinically relevant pre-clinical models. TILT-322 comprises a backbone of Ad5/3-E2F-d24[1,3] and carries a human anti-Mucin1 (aMUC1) and anti-CD3 (aCD3) T cell engager by linking single domain antibodies against MUC1 and CD3.[3]

MUC1 is a transmembrane glycoprotein which is aberrantly expressed in tumors and thus acts as a tumor-associated antigen in various types of cancer.[4] MUC1 dysregulation has been linked to tumor development, metastasis, and poor clinical outcomes.[5,6] The percentage of cancer patients expressing human MUC1 varies depending on the type of cancer and the stage of the disease. Up to 90% of breast tumors, approximately 70%–90% of pancreatic ductal adenocarcinomas and ovarian cancers (OvCas), 50%–80% of lung adenocarcinomas and colorectal cancer, and 30%–70% of prostate cancers reported to express abnormal MUC1 expression.[7] This makes MUC1 a potential candidate to use as a diagnostic and therapeutic target.

In addition, the TILT-322 construct features human IL-2, a classic T cell growth factor, which is expressed via an internal ribosome entry site (IRES). The aim of including IL-2 together with aMUC1aCD3 in

Received 17 November 2023; accepted 20 June 2024;
https://doi.org/10.1016/j.ymthe.2024.06.029.

**Correspondence:** Akseli Hemminki, Cancer Gene Therapy Group, Translational Immunology Research Program, Faculty of Medicine, University of Helsinki, Helsinki, Finland.
**E-mail:** akseli.hemminki@helsinki.fi

 © 2024 The Authors.
Published by Elsevier Inc. on behalf of The American Society of Gene and Cell Therapy.
This is an open access article under the CC BY license (http://creativecommons.org/licenses/by/4.0/).

TILT-322 is to generate anti-tumor immune responses by accelerating effector CD8$^+$ T and helper CD4$^+$ T cell proliferation in Mucin1 (MUC1)-positive tumors. Among the numerous interleukins developed for tumor therapy, IL-2, initially called T cell growth factor, holds a significant place as it was the first to receive approval from the U.S. Food and Drug Administration for the treatment of metastatic melanoma and renal cell carcinoma.[8,9] Multiple studies have demonstrated the safe delivery of IL-2 to the tumor microenvironment using oncolytic adenoviruses.[1,8] IL-2 has been widely used in the context of adoptive cell therapy.[1] However, IL-2 monotherapy has notable limitations, including a short serum half-life, low tumor accumulation before systemic toxicity limits dose, and life-threatening adverse events at high doses.[1,8] Therefore, the concept of tumor-specific production from an oncolytic vector platform presents an appealing solution.

The binding of aMUC1aCD3 bispecific T cell engager (BsTe) to its target cells triggers T cell activation leading to the formation of an immune synapse that bridges tumor and T cells.[3,10] BsTe is a type of immunotherapy designed to harness the power of the immune system by simultaneously binding to two different antigens. One arm of the BsTe molecule binds to a specific antigen present on the surface of cancer cells, while the other arm binds to the CD3 receptor on T cells leading to tumor destruction.[11]

However, the systemic delivery of recombinant BsTe has often resulted in adverse events in healthy organs before achieving a sufficiently high concentration in the tumor. This dilemma underscores the rationale for local production within the tumor as described here.[12] Upon continuous exposure to antigens, T cells start co-expressing a high level of inhibitory receptors including programmed cell death protein 1 (PD-1), T cell immunoglobulin and mucin domain 3 (TIM-3), tyrosine-based inhibitory motif domain (TIGIT), cytotoxic T lymphocyte antigen-4 (CTLA-4), lymphocyte activation gene 3 (LAG-3), and B- and T-lymphocyte attenuator (BTLA).[13] This phenomenon is referred to as T cell exhaustion and has been well documented in the context of chronic viral infection and cancer.[13] We aimed to study if T cell immunity could be generated and exhaustion prevented by TILT-322.[14] As substrates, we used highly clinically relevant OvCa ascites specimens and a humanized mouse model featuring a patient explant.

## RESULTS

### TILT-322 virus engages the response of unstimulated unmatched CD3 cells in infected cancer cells

TILT-322 is a chimeric virus mostly from serotype 5 but with a serotype 3 fiber knob (Figure 1A). The transgene cassette (aMUC1aCD3-IRES-IL-2) was inserted into the E3 region of the virus in such a way that transgene expression occurs exclusively when the virus replicates.[15] To achieve tumor-specific replication, the viral backbone contains a 24-bp deletion in the retinoblastoma binding region of adenoviral E1A together with the E2F promotor placed in front of the E1A region.[1] The replication of TILT-322 in human MUC1-expressing A549 cells supernatants is shown in Figure S1A. Furthermore, TILT-322 exhibited the ability to effectively target and kill a range of human cancer cells expressing MUC1, including A549, T47D, PDX-OvCa, MDAMB-231, and PC3MM2, with comparable efficacy to the unarmed backbone virus (Figures S1B–S1F). The therapeutic response obtained varies slightly due to the varying levels of MUC1 antigen expression on the cell surface. When TILT-322 was combined with allogenic human CD3$^+$ T cells, statistically significant cell-killing was observed compared to the tumor cells-only group in (PDX-OvCa at 96 h; $p$ = 0.0282), MDAMB-231 ($p$ = 0.0240) and PC3MM2 cells ($p$ = 0.0114) at 96 h after treatment as shown in Figures 1B–1D. The same effect was observed in A549 ($p$ = 0.0057) and T47D ($p$ = 0.016) cells at 120 h as demonstrated in Figures S2A and S2B. Moreover, we observed that the tumor cell cytotoxicity of the TILT-322 virus predominantly occurs in the presence of T cells in PDX-OvCa (Figure S2C), MDAMB-231 (Figure S2D) and PC3MM2 (Figure S2E) cells. However, no statistical difference was evident as the virus alone group was also capable of inducing a tumor-killing effect, suggesting an intriguing aspect of the viral intervention's mechanism.

Importantly, quantification of human IL-2 in the supernatant of TILT-322-infected A549 cells indicated the production of IL-2 from the aMUC1aCD3-IL-2 transgene *in vitro* (Figure 1E). This result also suggests the release of aMUC1aCD3-BsTe transgene following TILT-322 infection. To explore the effects of TILT-322 with human CD3$^+$ T cells and to provide a mechanistic insight into TILT-322 mediated tumor cell death *in vitro*, a panel of tumor cells was incubated with TILT-322 and CD3$^+$ T cells. A statistically significantly higher level of activated CD8$^+$ T cells via CD69$^+$ and CD25$^+$ expression was noted in the TILT-322 virus-treated group compared to Ad5/3-E2F-d24-aMUC1aCD3 + T cell, backbone + T cell and tumor + T cell groups (Figures 1F and 1G). CD69$^+$ and CD25$^+$ T cell expression upon TILT-322 infection of A549 and T47D in the presence of CD3$^+$ T cells is shown in Figures S2F–S2I.

Overall, these findings suggested that TILT-322 is replicative in human cancer cells and has a potent antitumor activity when paired with T cells and that it elicits T cell activation.

### TILT-322 stimulates lymphocyte activation in *ex vivo* cultures of patient-derived ascites

The mechanism of action of TILT-322 was investigated *ex vivo* in clinical ascites samples from human OvCa patients with peritoneal carcinomatosis (Table 1). Cell cytotoxicity assay performed on four different ascites patient samples showed TILT-322 virus-mediated enhanced cell killing in all tested samples (Figures 2A–2D). When TILT-322 kills MUC1-positive tumor cells, MUC1 expression is reduced.[3] Thus, the effects of TILT-322 infection can be seen in the lower expression of MUC1 antigen-expressing ascites cells and higher lymphocytes. All four tested ascites samples showed significantly reduced levels of overall MUC1-positive cells (Figures S3A–S3D), a positive marker for OvCa malignant cells[16] (ascites 1, $p$ = 0.001; ascites 2, $p$ = 0.01; ascites 3, $p$ = 0.001; and ascites 4, $p$ = 0.001) and a higher expression of CD3$^+$ (Figures S3E–S3H) T cells (ascites 1, $p$ = 0.0001; and ascites 3, $p$ = 0.05) after the TILT-322 virus treatment.

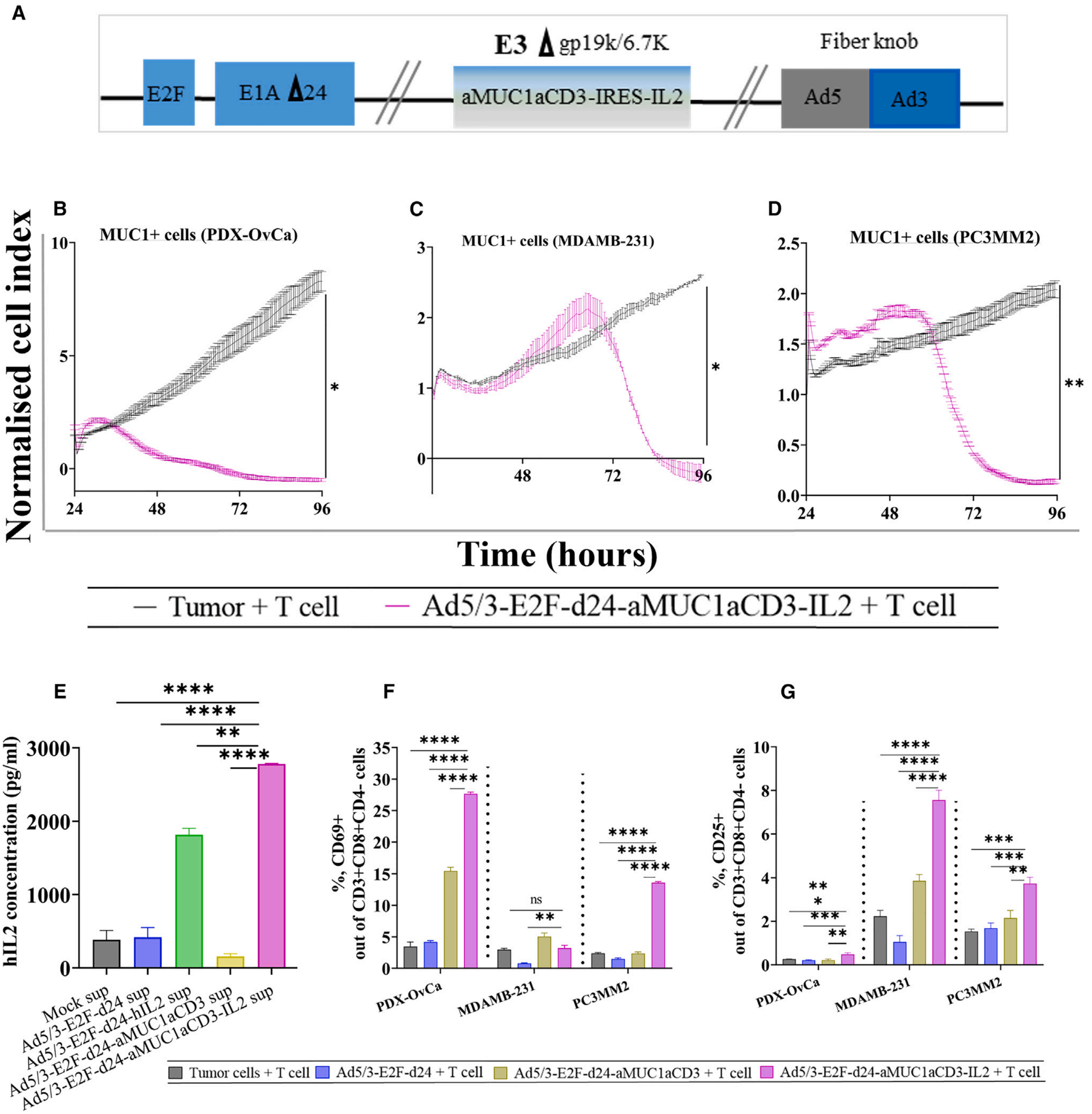


**Figure 1. Ad5/3-E2F-d24-aMUC1aCD3-IL2/TILT-322 schematic view and its oncolytic activity *in vitro***

(A) Schematic presentation of chimeric oncolytic adenovirus with E2F promoter; 24-base-pair deletion in E1A; aMUC1aCD3 IRES-IL2 inserted in the E3 region; and an Ad3 serotype knob in the Ad5 fiber.

(B–D) Real-time xCELLigence-based cytotoxicity assay showing the lysis of MUC1-positive PDX-OvCa (C) MDAMB-231 and, (D) PC3MM2 tumor cells in the presence of virus and T cells at E:T ratio of 1:1 for up to 120 h. The mean ± SEM of duplicates is shown.

(E) Level of IL2 in the infected cancer cell line (A549) supernatants collected after 48 h of infection. Effect of Ad5/3-E2F-d24-aMUC1aCD3-IL2 treatment on T cell activation.

(F and G) Increased activation of $CD69^+$ and, (G) $CD25^+$ out of $CD3^+CD4^-CD8^+$ cells cultured for 3 days at E:T ratio of 5:1 with virus and $MUC1^+$ tumor cells assessed by flow cytometry. Cells were collected, washed, stained with antibodies, and analyzed by flow cytometry afterward. All experiments were run in quadruplicates, and the resulting data are presented as mean ± SEM. The statistical difference among different groups of iCElligence assay was calculated by using Welch's t test and one-way ANOVA with Tukey's post hoc test was used to compare more than two groups in the activation assay. Statistical significance is represented as *$p < 0.05$, **$p < 0.01$, ***$p < 0.001$, and ****$p < 0.0001$. ns, non-significant.

**Table 1. Characteristics of OvCa patients used in this research**

| Sample ID | Age, y | Diagnosis | Initial tumor location | Metastases | Prior therapies (for cancer) |
|---|---|---|---|---|---|
| Ascites 1 | 59 | sigmoid adenocarcinoma | sigmoid colon | peritoneal carcinomatosis | none |
| Ascites 2 | 61 | High-grade serous ovarian | fallopian tube | peritoneum cavity | none |
| Ascites 3 | 68 | High-grade serous ovarian | fallopian tube | peritoneal carcinomatosis | none |
| Ascites 4 | 75 | High-grade serous ovarian | fallopian tube | peritoneal carcinomatosis | none |

To investigate TILT-322 virus-mediated lymphocyte activation in ascites samples, the levels of CD69$^+$ and CD25$^+$ cells were assessed. The total percentage of activated CD69$^+$CD8$^+$ T cells was statistically higher in all four tested samples when infected with TILT-322 and compared to respective control groups (Figure 2E). Similar results were observed when assessing the CD25 activation marker in CD8$^+$ T cells in all ascites samples (Figure 2F). The activity of these cells was further validated by the significant presence of cytotoxic molecules such as granzyme-B (Figure 2G) and interferon-gamma (Figure 2H) in CD8$^+$ T cells in TILT-322 treated groups.

Furthermore, we explored if TILT-322 could also impact natural killer (NK)-T (CD3$^+$, CD56$^+$) (Figures S3I–S3L) and NK (CD3$^-$, CD56$^+$) (Figures S3M–S3P) cells in the samples. Three out of four ascites samples showed a statistically higher percentage of the latter compared to the ascites cells only control group suggesting TILT-322 may also engage NK-T and NK cells to kill tumor cells.

Overall, these data demonstrated the ability of TILT-322 to kill cancer cells present in patient-derived ascites with increased activation of the lymphocyte population.

### TILT-322 activates gamma delta T cells in ovarian ascites samples

We investigated TILT-322-mediated activation of the gamma delta ($\gamma\delta$) T cell population using CD69 surface expression[17,18] and intracellular granzyme B, perforin, and interferon-gamma expression in ovarian ascites samples. To examine the activation of $\gamma\delta$ T cells, ovarian ascites samples were infected by the TILT-322 virus. Figures S4A–S4D shows the level of $\gamma\delta$ T cells in ascites cells followed by TILT-322 treatment. The data depicted in Figure 3A show the highest level of activated CD69$^+$ $\gamma\delta$ T cells in the TILT-322 treated group. Similarly, the levels of CD69$^+$ cells on V$\delta$1$^+$ and V$\delta$2$^+$ cell subsets are also high in TILT-322-treated groups as shown in Figures S4E–S4H. Furthermore, we analyzed the levels of cytotoxic cytokines such as granzyme-B (Figure 3B), perforin (Figure 3C), and interferon-gamma (Figures S4I–S4L), which are mostly significantly elevated in TILT-322-treated samples compared to controls.

### Investigation of T cell exhaustion in OvCa ascites

To investigate T cell exhaustion due to continuous T cell engagement, we examined the expression of inhibitory receptors (PD-1$^+$, TIM-3$^+$, TIGIT$^+$, LAG-3$^+$, BTLA$^+$, and CTLA4$^+$) in CD8$^+$ T cells (Figures 4A–4D). We observed a significant decrease in the expression of these inhibitory markers following TILT-322 treatment in all four ascites samples. To investigate the T cell exhaustion at the transcriptional level, two important markers such as TOX (thymocyte selection-associated HMG-box protein) and T cell factor 1 (TCF-1) expression were analyzed following TILT-322 treatment. In our study, we observed subtle variations in the therapeutic responses across different ascites samples. Our data reveal a notable reduction but not statistically significant level of TOX expression on CD4$^+$ (Figure S5A) and CD8$^+$ (Figure S5B) T cells following TILT-322 treatment, shedding light on the mechanism underlying the therapeutic efficacy of this novel immunotherapy. The finding reveals a substantial elevation in the percentage of TCF-1 on both CD4$^+$ (Figure S5C) and CD8$^+$ T (Figure S5D) cells after treatment, suggesting a potential mechanism by which TILT-322 lessens T cell exhaustion and fosters immune rejuvenation.

These findings suggested that TILT-322 has the potential to control exhausted T cells in ovarian ascites samples. Moreover, these findings underscore the therapeutic potential of TILT-322 in reversing T cell exhaustion and unleashing potent antitumor immune responses, thereby offering promising prospects for the development of more effective cancer immunotherapies.

### TILT-322 treatment in ovarian ascites samples downregulates genes related to tumor growth control

To gain further insights into the mechanism of action of TILT-322, RNA sequencing (RNA-seq) was performed on ovarian ascites samples treated with Ad5/3-E2F-d24 and TILT-322 viruses at 5 days after infection. TILT-322 infection of ovarian ascites samples led to downregulation of 14 differentially expressed genes (SLC26A4-AS1, APOE, TREM2, CD36, AC145676.1, VENTX, FP671120.4, AC007663.4, AC112220.2, C1QC, TEPP, AC027796.4, AC016026.1, and C1QA) in ascites samples (Figure S6A). Genes such as APOE, TREM2, VENTX, and C1QA are associated with tumor growth and progression, promotion of apoptosis, and inflammation. Genes AC145676.1, FP671120.4, AC112220.2, AC027796.4, and AC016020.1 were upregulated, but no pathways associated with these were found. These genes might be long non-coding RNAs.

Overall, these finding provides insight into the possible biological mechanism that might be involved in TILT-322-mediated tumor cell killing.

### Antitumor efficacy of TILT-322 *in vivo*

To characterize TILT-322 in a model that allows for the dynamics of the immune system, we tested the therapy in a clinically relevant patient-derived humanized xenograft (PDX) of OvCa in mice. The virus

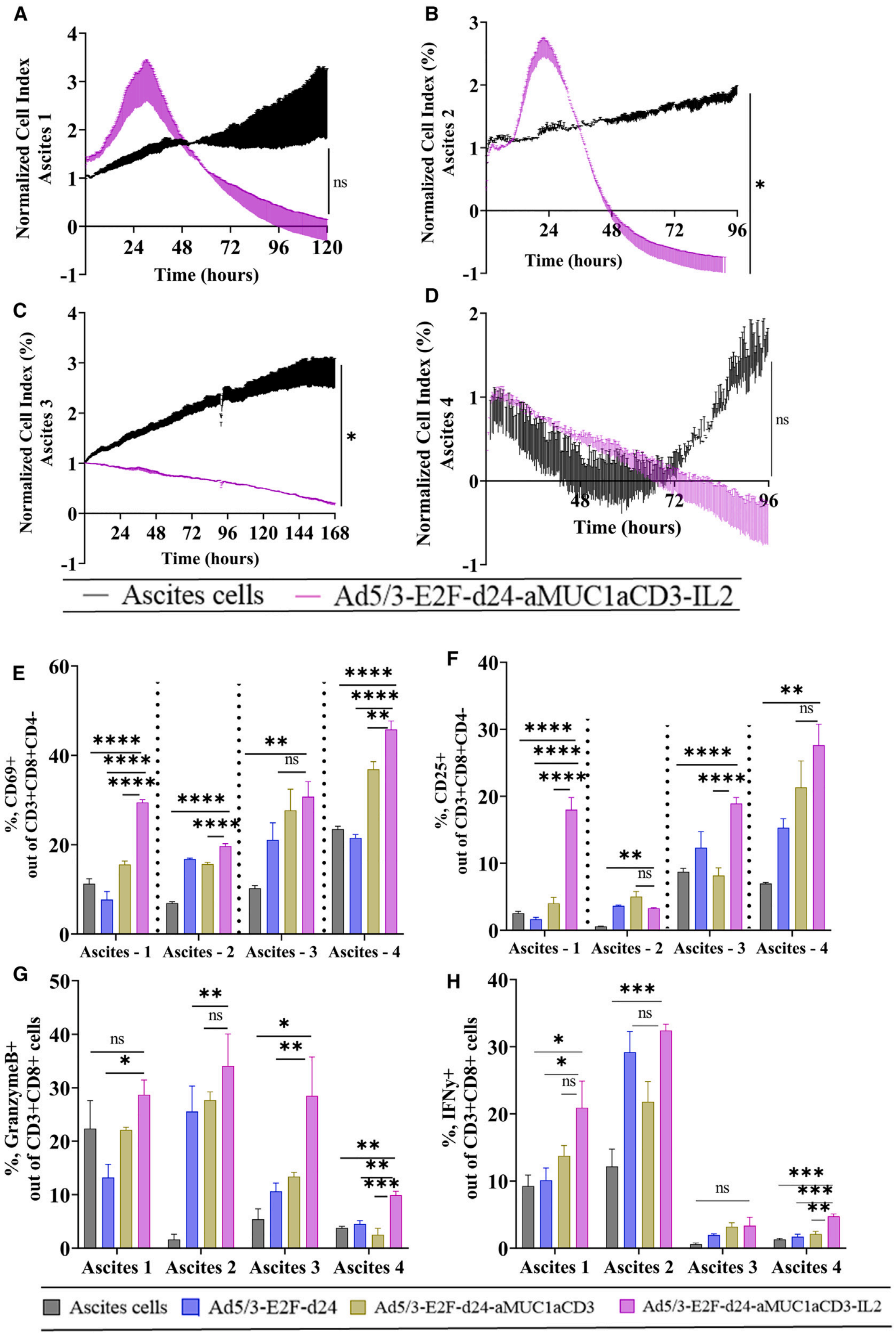


*(legend on next page)*

(legend on next page)

dosage used in this study is either directly adapted or slightly modified from previously published work.[19,20] Mice were administered with a virus via intratumoral ($1 \times 10^9$ VP/tumor) and systemic ($1 \times 10^{10}$ VP/animal intravenously) routes. All mice (*n* = 7 per group) received peripheral blood mononuclear cells (PBMCs) ($1 \times 10^7$) intraperitoneally by following the treatment schedule shown in Figure S7A to model the presence of a human immune system. When administered locally at the tumor site, TILT-322 demonstrated significantly improved tumor growth control compared to control groups (backbone virus-treated mice $^{***}p = 0.001$ and untreated mice/mock $^{****}p = 0.0001$) (Figure 5A). TILT-322 demonstrated a superior antitumor effect compared to Ad5/3-E2F-d24-aMUC1aCD3/TILT-321 virus-treated mice $^{****}p = 0.0001$ as shown in Figure 5A.

Importantly, significantly improved tumor growth control was evident when TILT-322 was administered intravenously compared to mock ($^{****}p = 0.0001$), as shown in Figure 5B.

Blood and tumor samples collected from animals euthanized on day 27 were analyzed to examine the circulating and infiltrating lymphocyte population respectively. Evidence of higher levels of $CD3^+$ T cells (Figure 5C) and $CD8^+$ T cells (Figure 5D) was observed following intratumoral administration of TILT-322 when compared to the mock control group. Further analysis of $CD3^+CD8^+$ cells (Figure 5E) and activated $CD3^+CD8^+CD69^+$ T cells (Figure 5F) in TILT-322 (intratumoral) treated tumors showed significantly higher percentage when compared to backbone virus and mock. T cell profiling of blood from mice receiving intravenous TILT-322 showed statistically different expression of CD3+ ($^{***}p = 0.001$) (Figure 5G) and $CD8^+$ ($^{*}p = 0.05$) (Figure 5H) T cells when compared to the mock group. The result was similar when we investigated the activated lymphocyte population in TILT-322 (intravenous)-treated tumors (Figures 5I and 5J). Accompanying the reduced tumor volume seen in mice tumors treated with TILT-322 was the reduced expression of MUC1, which was evident in both intratumoral (Figure S7B) and intravenous (Figure S7C). We further investigated the expression levels of CD127, T-bet, and EOMES on T cells to obtain information regarding T cell differentiation status, effector functions, and potential for antitumor efficacy. The percentage of CD127 (Figures S7D and S7E) on $CD4^+$ and $CD8^+$ T cells in the TILT-322 (i.t.) treated group was statistically significant compared to backbone and PBMC controls, suggesting a favorable prognosis and enhanced antitumor immune responses. Furthermore, a noticeably higher percentage of T-bet cells on $CD4^+$ and $CD8^+$ T cells (Figures S7F and S7G) were obtained followed by the TILT-322 (i.t.) treatment. A similar result was obtained while accessing the total percentage of EOMES cells (Figures S7H and S7I). When TILT-322 was administered systemically, the total percentage of CD127 (Figures S7J and S7K), T-bet (Figures S7L and S7M), and EOMES (Figures S7N and S7O) on $CD4^+$ and $CD8^+$ T cells appeared visibly higher but not statistically significant in the majority of samples compared to the PBMC control group.

Overall, these data show that TILT-322 provides antitumor effects *in vivo* regardless of the administration route (either locally or intravenously), which is associated with T cell activity.

## DISCUSSION

In this research, we constructed a novel oncolytic adenovirus (TILT-322), armed with human aMUC1-aCD3-IL-2. We characterized TILT-322 *in vitro* using commercial tumor cells, *ex vivo* OvCa patient ascites, and *in vivo* in a humanized mouse model bearing a patient xenograft tumor. Both *in vitro* and *ex vivo*, TILT-322 showed the ability to increase T cell activity and induce the release of cytolytic agents such as granzyme B and perforin. In this study, the oncolytic effect of TILT-322 virus was investigated using a panel of different MUC1-expressing cells. Of note, the virus replicates in tumor cells that are defective in the Rb/p16 pathway and therefore oncolysis is not dependent on MUC1 expression. Instead, the role of the BsTe is to recruit and activate T cells against MUC1-positive tumor cells. Additionally, the transduction of tissues by the virus is determined by the interaction of the fiber knob with receptors such as desmoglein 2, not by MUC1. The adapter protein coded by the virus helps T cells recognize MUC1-positive tumor cells.

*In vivo*, TILT-322 treatment led to improved tumor control due to an increased activation of $CD8^+$ T cells in PDX-OvCa mice. Both local and intravenous TILT-322 treatment resulted in anti-tumor efficacy in the presence of human PBMCs. Depending on the level of MUC1 antigen expression, the therapeutic response of TILT-322 was slightly different among different tumors and patient samples.

To provide mechanistic insight into the TILT-322 mechanism of action, OvCa ascites were used as an *ex vivo* model. Ascites is an accumulation of fluid in the peritoneal cavity caused by the intraperitoneal spread of cancer.[21,22] It contains a mixture of tumor cells, noncancerous cells, and soluble substances.[22] Noncancerous cells in ascites include cancer-associated fibroblasts, mesenchymal stem cells, myeloid-derived suppressor cells, M2-like macrophages, and T lymphocytes, all of which influence tumor cell activity and responsiveness to therapy.[22] Thus, ascites serves as a good model to investigate the

**Figure 2. TILT-322 virus exerts an improved cytotoxic effect in OvCa ascites samples and stimulates T lymphocyte activation**

(A–D) Ascites cells were seeded using Matrigel and infected with the virus. Real-time xCELLigence-based cytotoxicity assay showing TILT-322 mediated killing of tumor cells that are present in (A) ascites 1, (B) ascites 2, (C) ascites 3, and (D) ascites 4 patient samples.

(E) Increased levels of positive $CD69^+$ and (F) $CD25^+$ T cells out of $CD3^+CD8^+CD4^-$ obtained after treating ascites samples with TILT-322 at 500 VP/cell for 8 days.

(G) Detection of increased expression of granzyme B-positive cells in $CD8^+$ T cells.

(H) Higher expression of interferon-gamma positive cells presents in $CD8^+$ T cells followed by TILT-322 infection. The resulting data are presented as mean ± SEM of quadruplets. One-way ANOVA with Tukey's post hoc test was used to run statistical significance and is represented as $^{*}p < 0.05$, $^{***}p < 0.001$, $^{****}p < 0.0001$. ns, non-significant.

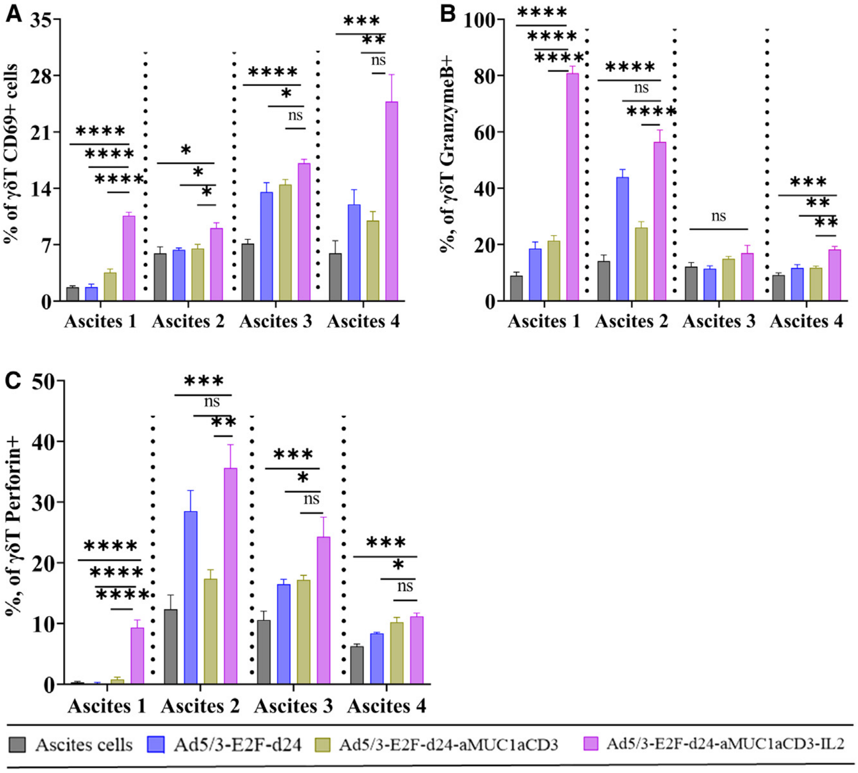


**Figure 3. Activation of γδ T cells present in ascites samples by TILT-322 treatment**

The graph shows the percentage of (A) CD69+ γδ T cells (B) granzyme B + γδ T cells, and (C) perforin + γδ T cells in four different ascites samples. Ascites cells were incubated with TILT-322 virus at 1,000 VP for 7 days followed by the cells staining for flow cytometry analysis. The resulting data are presented as mean ± SEM of quadruplet. Statistical significance was run using One-way ANOVA with Tukey's post hoc test and is represented as *$p < 0.05$, **$p < 0.01$, ***$p < 0.001$, ****$p < 0.0001$. ns, non-significant.

*ex vivo* functionality of TILT-322. In general, therapeutic agents like TILT-322 are engineered to target and activate T cells selectively without causing significant harm to normal, healthy T cells. Reduction in the T cell exhaustion and increased activation of T cells evaluated by different experimental conditions in this preclinical study suggests that TILT-322 functionality does not affect the viability of healthy T cells. Future research could explore which T cell clones exhibit a higher propensity for activation by the Ad5/3-E2F-d24-aMUC1aCD3-IL2 virus. This investigation will also yield insights into T cell viability, exhaustion, and their potential involvement in the reprogramming process. Additionally, in future experiments, including a viability dye in human samples could enhance resolution and lead to precise, reliable data interpretation of flow cytometry assay.

In this preclinical study, the *in vivo* anticancer activity of TILT-322 was assessed utilizing an *in vivo* tumor model. We believe that human data on patient-derived ascites samples is even more helpful for gaining direct insights into the complexities of human diseases and treatment responses, by avoiding the constraints inherent in animal models. Yet, to evaluate the efficacy of a therapeutic intervention, future studies could use more than one *in vivo* tumor model. Different tumor models may have diverse properties, hence employing many tumor models improves the robustness of results.

Previous studies have reported that ascites in OvCa are immunosuppressive and consist of abundant amounts of CTLA-4 as a part of the suppressive mechanism.[23] Another study reported the presence of PD-1 expression in ovarian ascites-derived T cells.[24] These suppressive factors identified in ascites samples that reduce T cell functions were reversed by the effects of TILT-322 treatment in T cell subsets. TILT-322 treatment of ovarian ascites resulted in a trend toward a lower expression of inhibitory molecules such as PD-1, LAG-3, TIGIT, TIM-3, CTLA4, and BTLA. Other published studies[25,26] reported that the inhibition of these molecules is associated with prognostically significant biomarkers and potential immunotherapies to treat cancer in the clinic. High levels of TCF-1 expression have been linked to better antitumor responses and longer survival in cancer patients. TCF-1-positive T cells have increased proliferative capacity and maintained effector function, which contributes to their ability to provide efficient immune surveillance against tumor cells.[27] TCF-1 is critical for suppressing the transcriptional pathways that cause terminal T cell exhaustion. It competes with other transcription factors, such as TOX and Blimp-1, which regulate the expression of exhaustion-associated genes and promote the development of terminally exhausted T cell subsets.[27,28]

TILT-322 was able to induce cytotoxicity of CD8+ T cells, NK cells, NK-like T cells, and γδ T cells to drive killing of MUC1 expressing tumor cells. TILT-322 treatment might influence the expansion or activation of NK-T and NK cells evidenced by its increased number followed by the virus infection. Future studies could be performed to explore the specific impact of TILT-322 on this interaction. Understanding the ability of NK-T and NK cells to engage with and target tumor cells provides crucial insight for evaluating the potential effectiveness of TILT-322 as an immunotherapy agent.

γδ T cells can be redirected by T cell engagers due to their unique features, such as their inherent anti-tumor properties, ability to act like antigen-presenting cells, and the advantage of having no major histocompatibility complex restrictions, allowing for greater flexibility in their utility to target tumors, compared to their αβ T cell counterpart.[29] Our study supports the finding of previous work which suggested that a bispecific antibody can simultaneously bind to the

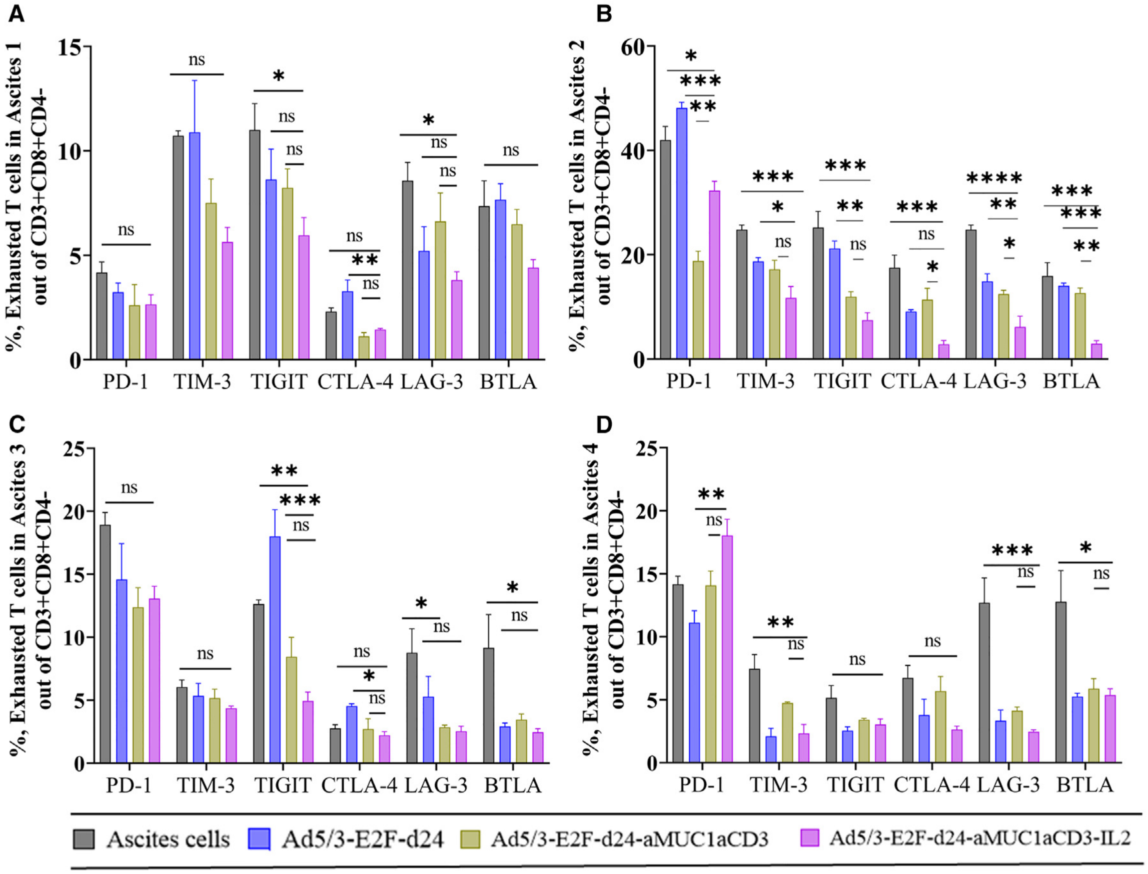


**Figure 4. Reduction of T cell exhaustion in ovarian ascites followed by TILT-322 treatment**

Ovarian ascites samples were treated with the TILT-322 virus for 5 days. Cells were then stained and analyzed by flow cytometry to access the expression of exhausted T cells. Percentage of Expression of inhibitory receptors such as PD-1$^+$, TIM-3$^+$, TIGIT$^+$, CTLA-4$^+$, LAG-3$^+$, and BTLA$^+$ cells (A–D) out of CD3$^+$CD8$^+$CD4$^-$ T cell. The resulting data are presented as mean ± SEM of quadruplet. One-way ANOVA with Tukey's post hoc test was run to determine statistical significance and the result is represented as ***$p < 0.001$, ****$p < 0.0001$. ns, non-significant.

Vγ9 chain of the Vγ9Vδ2$^+$ γδ T cell receptor and can selectively activate Vγ9$^+$ γδ T cells as judged by CD69 surface expression, and intracellular Granzyme B expression.[17] However, we believe that the discovery of other activation markers than CD69 in terms of γδ T cell study could serve as a better marker to access the activation of γδ T cells. Functional studies assessing the cytotoxic activity of γδ T cells in the presence of TILT-322 would provide valuable insights into the mechanism of action of this therapeutic agent and its potential efficacy in cancer immunotherapy in the future.

Tumor site expression of MUC1 often correlates with disease progression across a wide range of cancer types.[4,30] TILT-322 treatment of ovarian ascites cells showed a decrease in MUC1 levels, correlating with a positive therapeutic effect. In future studies, it is worth investigating the biological pathways and downstream signaling more to better understand this result. RNA-Seq result demonstrated the downregulation of apolipoprotein E plays a key role in controlling tumorigenesis and progression, such as cell proliferation, angiogenesis, and metastasis.[31] Hemopoietic progenitor homeobox protein/VENTX downregulation is essential for normal T cell development.[32] TREM2 is highly expressed in esophageal carcinoma cells and directly maintains their survival. Downregulation of TREM2 in renal cell carcinoma blocks the G1 phase and promotes apoptosis of tumor cells, and thus inhibits tumor development.[33] C1QA is expressed in the stroma and vascular endothelium of several human malignant tumors and its downregulation results in improved therapeutic control by controlling tumor metastasis.[34] RNA-seq of human ascites cells infected by oncolytic adenovirus such as TILT-322 represent a crucial step toward enhancing our understanding and refining the treatment of OvCa using oncolytic adenoviruses like TILT-322. This analysis not only deepens our understanding of the mechanisms underlying oncolytic viral therapy but also lays the groundwork for future improvements in treatment strategies tailored to the unique challenges posed by OvCa.

Considering recent findings suggesting increased survival in OvCa patients with γδ T cell infiltration into tumors,[35] we were interested in analyzing the effect of TILT-322 treatment on these cells. We found

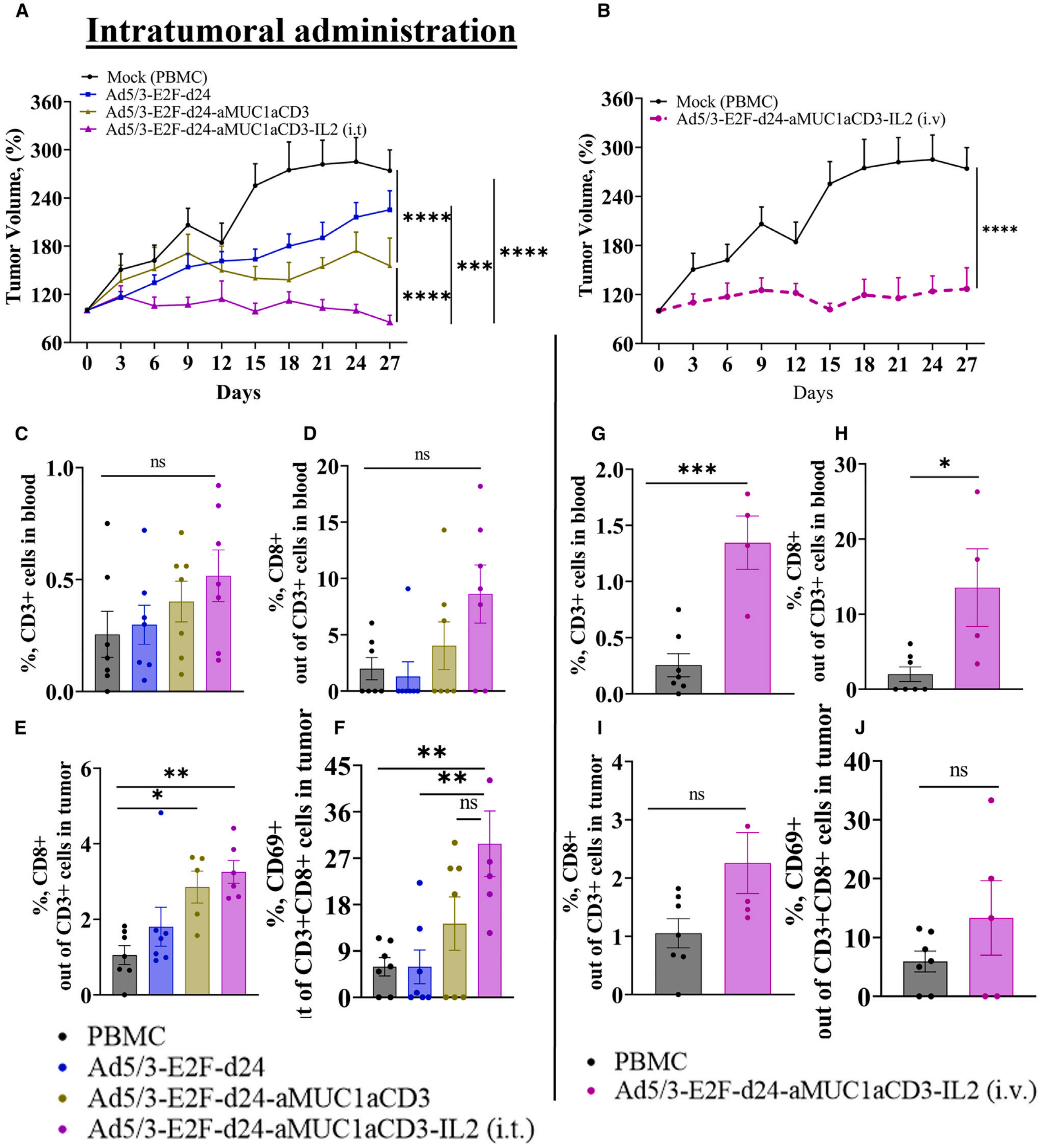


**Figure 5. *In vivo* efficacy of Ad5/3-E2F-d24-aMUC1aCD3-IL2 virus in an OvCa PDX model**

(A–D) (A) Enhanced anti-tumor efficacy of TILT-322 achieved by the intratumoral and (B) intravenous administration *in vivo*. Tumor volumes were measured every day until day 27 and the tumor growth curve was generated by normalizing against day 0 tumor volume. PDX-OvCa tumors were implanted subcutaneously in 4-week-old immuno-deficient female mice. PBMCs were administered intraperitoneally, and viruses were injected either intratumorally or intravenously. The mock (PBMC) group received an intratumor injection of PBS during treatment days. On the day of euthanization, mice blood and tumor samples were collected and stained for flow cytometry. Immune cell analysis of blood samples from mice receiving intratumoral treatment showing positive percentage of (C) $CD3^+$ and, (D) $CD8^+$ T cells.

*(legend continued on next page)*

that TILT-322 treatment led to increased levels of activated CD69$^+$ γδ T cell and higher expression of granzyme B and perforin.

The memory response is desirable in cancer immunotherapy because it provides long-term immune monitoring and protects against tumor recurrence.[36] When evaluating tumors treated with TILT-322, an increase in the percentage of CD127-expressing T cells inside the tumor microenvironment may indicate the formation of memory T cell populations. This suggests that the therapy may induce a durable immune response against tumor-specific antigens. Future studies may consider incorporating additional markers related to memory formation, and tumor infiltration to better elucidate the immune response dynamics following TILT-322 treatment. Despite the favorable attributes of IL-2, including its potent stimulation of T cell activation and effector functions,[37] the further iteration of this strategy may involve substituting IL-2 with another cytokine, such as IL-7,[19] to explore its potential synergistic effects and enhance therapeutic outcomes.

Taken together, our data suggest that TILT-322 is an attractive immunotherapeutic approach due to its ability to increase T cell activation *in vitro*, slow down T cell exhaustion *ex vivo*, and improve tumor growth control *in vivo*. These preclinical characteristics make it a promising candidate for future testing in clinical trials.

## MATERIALS AND METHODS

### Cell lines

The human cancer cell lines A549 (lung adenocarcinoma), HEK-293 (human embryonic kidney), T47D (breast epithelial adenocarcinoma), and MDAMB-231 (breast epithelial adenocarcinoma) were purchased from the American Type Culture Collection. All cell lines were cultured under the recommended conditions. PC3MM2 (prostate cancer) was gifted by Isaiah J. Fidler, M.D. Anderson Cancer Center, and maintained as described previously.[38] PDX-OvCa cells were developed as described earlier.[19]

### Human PBMC expansion and isolation

PBMCs were isolated from the buffy coat of a healthy donor (Finnish Red Cross Blood) using lymphoprep gradient density (StemCell Technologies) followed by PBS wash (Sigma) and erythrocytes lysis with ammonium-chloride-potassium (ACK) red blood cell lysis buffer (Sigma). A Human Pan T isolation kit (130-096-535, Miltenyi Biotec) was used to negatively isolate CD3 T cells following the manufacturer's instructions. Patient blood was used to isolate autologous PBMCs and expanded in a similar way as described.[19]

### Virus construct

All viruses studied shared the Ad5/3-E2F-d24 backbone structure.[39] The virus construct (TILT-321) coding for aMUC1aCD3-BsTe has been described previously.[3] To generate TILT-322 (https://patents.google.com/patent/US11485791B2/en), a human IL-2 coding sequence was inserted in addition to aMUC1aCD3-BsTe connected by an IRES under E3 gene promotor region by replacing gp19k and 6.7k, as described before.[1]

### Cell viability assay

For the cytotoxicity study, a panel of MUC1-expressing human cell lines A549, T47D, PDX-OvCa, MDAMB-231, and PC3MM2 were used. We seeded 10,000 cells/well on 96-well plates (flat bottom) for 24 h before virus infection with Ad5/3-E2F-d24 (also referred to as a backbone or unarmed backbone), Ad5/3-E2F-d24-hIL-2, and TILT-322 at doses of 10, 100, 1,000, or 10,000 VP/cell. The assay was performed in quadruplet. Cell viability was determined on day 3 (A549, PDX-OvCa, and MDAMB-231), day 5 (PC3MM2), and day 7 (T47D) using CellTiter 96 Aqueous One Solution Proliferation Assay reagent (Promega) according to the manufacturers' instructions. The absorbance was read at 490 nm using a Hidex Sense plate reader (Hidex). The data were normalized to those of the uninfected control group.

T cell-mediated enhanced tumor cell killing by TILT-322 was assessed by MTS and iCELLigence Real-Time Cell Analyzer (RTCA) (Agilent Technologies).

In the iCELLigence assay, a total of 10,000 cancer cells per well were seeded and infected with Ad5/3-E2F-d24 and TILT-322 virus after 24 h at 100 VP/cell using and effector to target (E:T) ratio of 1:1. Unstimulated CD3 T cells were used as effector cells. In the MTS assay, a monolayer of MUC1-positive cells was infected with TILT-322 virus with or without T cells at a virus concentration of 1 pfu/cell using an E:T ratio of 5. On day 2 after infection, wells were gently washed twice with 100 μL PBS. Cell viability was measured using 20% of CellTiter 96 AQueous One Solution Proliferation Assay reagent, as described above.

In an *ex vivo* killing assay, 30,000 cells derived from OvCa ascites patient samples were seeded and infected with Ad5/3-E2F-d24 or TILT-322 virus at 500 VP/cell concentration. E-plates were coated with Matrigel at a concentration of 1 mg/mL followed by 1 h of incubation.

(E and F) (E) CD8$^+$ and (F) CD8$^+$CD69$^+$ T cells in tumor samples from mice receiving intratumoral treatment.
(G–H) Similarly, T cell expression in blood samples from mice receiving intravenous treatment is shown. Activated tumor infiltrating (I) CD8$^+$ and, (J) CD8$^+$CD69$^+$ lymphocytes in tumor (intravenous) were assessed by flow cytometry. The resulting data are presented as mean ± SEM ($n$ = 7, per group). One-way ANOVA with Tukey's post hoc test was used to run statistical significance of flow cytometry results. The result is represented as *$p$ < 0.05, **$p$ = 0.01, ***$p$ < 0.001, ****$p$ < 0.0001. ns, non-significant. Tumor growth curves were compared using a two-way ANOVA mixed-model analysis with the Tukey multiple comparisons test. Results were considered statistically significant at $p$ < 0.05. Data are presented as mean ± standard error of the mean (SEM).

Cell impedance values were monitored every 15 min for 120 h, and results were presented as normalized cell index values. The xCELLigence RTCA Agilent Technologies software (version 1.0) was used for data analysis.

### IL-2 detection by ELISA

MUC1-expressing A549 cells were infected with Ad5/3-E2F-d24, Ad5/3-E2F-d24-hIL-2, Ad5/3-E2F-d24-aMUC1aCD3, and TILT-322 virus using a concentration of 100 pfu/cell. Uninfected cells were used as Mock control. Supernatant from infected cells was collected after 48 h and filtered using 100-kDa and 10-kDa filters to eliminate more than 100 and more than 10 kDa, respectively. Human IL-2 was detected from filtered cell supernatants using Invitrogen Human IL-2 ELISA Kit (Human IL-2 ELISA Kit, Thermo Fisher Scientific) according to the manufacturer's instructions. Absorbance was measured at 490 nm using a Hidex Sense spectrophotometer.

### qPCR

A monolayer of A549 cells was infected with Ad5/3-E2F-d24 and TILT-322 viruses at a concentration of 1 pfu/cell. After 24 h of incubation, cells were washed twice with PBS followed by further incubation for 48 h. Supernatants was harvested at 48 h. DNA from supernatants was extracted using a High Pure Viral Nucleic Acid Kit (Roche) following the manufacturer's instructions. E1A gene copy number was quantified as a measure of virus replication and the total gene expression was normalized to that of the hBactin housekeeping gene, as described previously.[3]

### Collection and processing of ascites

Ascites human samples derived from OvCa were collected from three patients undergoing surgical resection. Ascites fluids were processed using a protocol previously published.[22] Briefly, freshly collected ascites fluid was received in a sterile drainage bag. Ascites fluids were transferred to a sterile 50-mL falcon tube for centrifugation at 400×*g* for 10 min. Erythrocytes were removed by ACK red blood cell lysis buffer (Sigma) from the cell pellet followed by cell washing with PBS. Cells were stored at −140°C using 10% DMSO-containing freezing media for long-term storage.

### T cell activation assay

The monolayer of a panel of MUC1 positive tumor cells (A549, T47D, PDX-OvCa, MDAMB-231, and PC3MM2) was co-cultured with 50,000 unmatched unstimulated $CD3^+$ T cells per well at a 5:1 ratio, followed by 3 days of incubation. For the *ex vivo* activation assay, 350,000 ascites cells were seeded in quadruplets and infected with the virus (Ad5/3-E2F-d24 or TILT-322). TILT-322 mediated γδ T cells activation in ascites was assessed using the same conditions assay, as described above.

Cells were collected after 7 days of incubation and stained with antibodies for flow cytometry. A list of antibodies used is provided in Table S1.

### Evaluation of T cell exhaustion profile in cell fraction of ascites human samples

In a 96-well plate (U-bottom), 350,000 ascites cells were seeded in quadruplicate. After 5 days of incubation, the cells were infected with Ad5/3-E2F-d24 and TILT-322 viruses. After 5 days of incubation, cells were collected and stained with antibodies for flow cytometry. A total of 100,000 events were collected and the list of antibodies used is mentioned in Table S1.

### Flow cytometry analysis

For T cell activation assays, cells were stained with human anti (a) CD3 (FITC, OKT3, BioLegend), aCD4 (AF700, A161A1, BioLegend), aCD8 (BV421, SK1, BioLegend), aCD69 (PE-Cy7, FN50, BioLegend), and aCD25 (PE-Cy5, M-A251, BioLegend) antibodies were used to run flow cytometry.

In the *ex vivo* assay, ascites cells were stained with human aCD3 (FITC, OKT3, BioLegend), aCD4 (AF700, A161A1, BioLegend), aCD8 (BV421, SK1, BioLegend), aCD69 (PE-Cy7, FN50, BioLegend), and aCD25 (PE-Cy5, M-A251, BioLegend) antibodies to analyze activated T cells. MUC1 expression was analyzed by using human aMUC1 (APC, 16A, BioLegend) antibodies in ascites. Human aCD56 (BV605, HCD56, BioLegend) antibody was used to check NK and NK-T cells.

For extracellular profiling of γδ T cell in ascites, human aCD3 (PE-CF594, UCHT1, BD sciences), TCR γδ (PE, 11F2, Miltenyi), aVδ1 (FITC, REA173, Miltenyi), aVδ2 (APC, REA771, Miltenyi), and aCD69 (PE-Cy7, FN50, BioLegend) antibodies were used. In intracellular staining antibodies such as Perforin (A-700, B-D48, Miltenyi), granzyme B (BV421, GB11, BD), and interferon-gamma (BV650, 4S.B3, BioLegend) were used. BD Cytofix/Cytoperm Plus Kit (with BD GolgiPlug) (BD Biosciences) was used for intracellular staining adapting manufacturer's instructions. Samples were stained after Fc blocking using the Human TruStain FcX Receptor Blocking Solution (BioLegend).

Immune analysis of the exhausted T cell panel in ascites consists of human antibodies such as aCD3 (FITC, OKT3, BioLegend), aCD4 (AF700, A161A1, BioLegend), aCD8 (BV421, SK1, BioLegend), BV-510 (SK1, BD sciences), PD-1 (PE-CF594, NAT105, BioLegend), TIM-3 (PE, A18087E, BioLegend), TIGIT (BV605, A15153G, BioLegend), LAG-3 (PE-Cy5, 11C3C65, BioLegend), BTLA (PE-Cy7, MIH26, BioLegend), CTLA4 (APC, BNI3, BioLegend), transcription factor TOX (PE, TXRX10, Invitrogen), and TCF-1 (BV-421, S33-966, BD).

To analyze the expression of MUC1, human aMUC1 (APC, 16A, BioLegend) was used. Immunological analysis of tumors derived from the humanized xenograft mouse model was performed using a similar panel as in the T cell activation assay. Markers such as CD127 (PE-CF594, HIL-7R-M21, BD Sciences), Tbet (APC, 4B10, BioLegend), and EOMES (PE, X4-83, BD Sciences) were used to

access additional roles of T cells in orchestrating effective immune responses against tumors.

NovoCyte Quanteon and NovoSampler Q System Bundle Flow Cytometry analyzer (Agilent) were used to run flow cytometry. All samples were acquired in quadruplets and 100,000 events were collected per sample. All fluorochromes used in each panel were compensated using the NovoSampler Q compensation matrix to prevent spillover leading to false positives. Cell data processing and gating were performed using FlowJo v.10.6.1 (Ashland).

### RNA-seq

RNA was extracted from ovarian ascites infected with Ad5/3-E2F-d24 and TILT-322 virus using RNAqueous Micro Total RNA Isolation Kit (Thermo Fisher Scientific) by following the manufacturer's instructions. Qubit4 Fluorometer was used to measure RNA concentration. Sequencing and analysis were performed by GENEWIZ using PolyA selection and 20–30 million reads per sample. Trimmomatic v.0.36 was used to trim sequence reads to remove any potential adaptor sequences and low-quality nucleotides. The trimmed reads were mapped to the Mus musculus GRCm38 reference genome, which is accessible on ENSEMBL, using the STAR aligner version 2.5.2b. Using featurCounts from the Subread package v.1.5.2, we computed the number of unique gene hits. The hit counts were summed and reported using the annotation file's gene_id feature. Unique readings located within exon regions were counted. If a strand-specific library preparation was carried out, the reads were counted accordingly. DESeq2 was used to compare gene expression between groups. The distribution of read counts in libraries was analyzed before and after normalization. The original read counts were normalized to account for factors such as differences in sequencing yield between samples. The normalized read counts were used to reliably identify differentially expressed genes. The Wald test was applied to calculate *p* values and log2 fold changes. Genes having an adjusted *p* values of less than 0.05 were labeled as differentially expressed.[15]

### Animal experiment

Four-week-old immunodeficient NOD/SCID/IL-2rg$^{-/-}$ mice (Jackson Laboratory) were injected subcutaneously with $5 \times 10^6$ PDX-OvCa cells. Mice were randomized into groups ($n = 7$) when tumors became injectable after 15 days of implantation. All mice received $10 \times 10^6$ autologous PBMC intraperitoneally two days before virus administration. Virus ($1 \times 10^9$ VP/tumor) or PBS was administered intratumorally, and one group received TILT-322 ($1 \times 10^{10}$ VP/tumor) intravenously every 3 days. Tumors were measured using an electronic caliper until day 27. Mice were euthanized afterward, and samples were collected. Tumor volume was estimated with a formula: $0.52 \times (\text{max dimension}) \times (\text{min dimension})^2$.

### Statistical analysis

For statistical analysis and graphical representation of the data, GraphPad Prism v.9.2.0 (GraphPad Software) was used. One-way ANOVA with Tukey's post hoc test was used to compare more than two groups of *in vitro* assays. The statistical difference among different groups of iCELLigence assay was calculated by using Welch's t test. Tumor growth curves were compared using a mixed-model analysis with the Tukey multiple comparisons test. Results were considered statistically significant at a *p* value of less than 0.05. Data are presented as mean ± SEM.

## DATA AND CODE AVAILABILITY

All data are available upon request.

## SUPPLEMENTAL INFORMATION

Supplemental information can be found online at https://doi.org/10.1016/j.ymthe.2024.06.029.

## ACKNOWLEDGMENTS

We thank Minna Oksanen and Sini Raatikainen for expert experimental and administrative assistance. Part of the work was carried out with the support of the HiLIFE Laboratory Animal Center Core Facility, University of Helsinki, Finland. We also thank the Biomedicum Flow Cytometry Unit (University of Helsinki, Helsinki, Finland). The Provincial Government of Southern Finland and the Experimental Animal Committee of the University of Helsinki (license number ESAVI/26562/2022) have approved all experiments described in the paper. The HUS Regional Committee on Medical Research Ethics (HUS/3360/2019) has evaluated and approved the project associated with patient samples. This study was supported by the Jane and Aatos Erkko Foundation, HUCH Research Funds (VTR), Finnish Cancer Organizations, University of Helsinki, TILT Biotherapeutics Ltd., Sigrid Jusélius Foundation, Novo Nordisk Foundation, Päivikki and Sakari Sohlberg Foundation, and the Finnish Red Cross Blood Service. This study received funding from the European Union's Horizon 2020 research and innovation program under the Marie Skłodowska-Curie grant agreements (No. 813453). We thank the Orion Foundation, Ida Montin Foundation, K. Albin Johansson Foundation, Selma and Maja-Lisa Selander's Fund in Research in Odontology (Minerva Foundation), Paulo Foundation, and the Finnish Cultural Foundation for grant support. This program was supported by an educational grant from the Gilead Nordic Fellowship Program. We thank Albert Ehrnrooth and Karl Fazer for their research support.

## AUTHOR CONTRIBUTIONS

S.B., M.V.H., D.Q., J.S., V.C.-C., and A.H. designed the experiments. S.B., M.V.H., E.J., S.V.K., L.H., V.A., and J.C. conducted the experiments. S.B., M.V.H., and A.H. analyzed the results. All authors contributed to the writing and reviewing of the manuscript.

## DECLARATION OF INTERESTS

A.H. is a shareholder of Circio Holdings ASA. (Norway). A.H., J.C., J.M.S., and D.Q. are employees and shareholders of TILT Biotherapeutics, Ltd.

## REFERENCES

1. Havunen, R., Siurala, M., Sorsa, S., Grönberg-Vähä-Koskela, S., Behr, M., Tähtinen, S., Santos, J.M., Karell, P., Rusanen, J., Nettelbeck, D.M., et al. (2017). Oncolytic

Adenoviruses Armed with Tumor Necrosis Factor Alpha and Interleukin-2 Enable Successful Adoptive Cell Therapy. Mol. Ther. Oncolytics *4*, 77–86. https://doi.org/10.1016/j.omto.2016.12.004.

2. Liikanen, I., Basnet, S., Quixabeira, D.C.A., Taipale, K., Hemminki, O., Oksanen, M., Kankainen, M., Juhila, J., Kanerva, A., Joensuu, T., et al. (2022). Oncolytic adenovirus decreases the proportion of TIM-3 + subset of tumor-infiltrating CD8 + T cells with correlation to improved survival in patients with cancer. J. Immunother. Cancer *10*, e003490. https://doi.org/10.1136/jitc-2021-003490.

3. Basnet, S., Santos, J.M., Quixabeira, D.C.A., Clubb, J.H.A., Grönberg-Vähä-Koskela, S.A.M., Arias, V., Pakola, S., Kudling, T.V., Heiniö, C., Havunen, R., et al. (2023). Oncolytic adenovirus coding for bispecific T cell engager against human MUC-1 potentiates T cell response against solid tumors. Mol. Ther. Oncolytics *28*, 59–73. https://doi.org/10.1016/j.omto.2022.12.007.

4. Acres, B., and Limacher, J.-M. (2005). MUC1 as a target antigen for cancer immunotherapy. Expert Rev. Vaccin. *4*, 493–502. https://doi.org/10.1586/14760584.4.4.493.

5. Jing, X., Liang, H., Hao, C., Yang, X., and Cui, X. (2019). Overexpression of MUC1 predicts poor prognosis in patients with breast cancer. Oncol. Rep. *41*, 801–810. https://doi.org/10.3892/or.2018.6887.

6. Chen, W., Zhang, Z., Zhang, S., Zhu, P., Ko, J.K.S., and Yung, K.K.L. (2021). Muc1: Structure, function, and clinic application in epithelial cancers. Int. J. Mol. Sci. *22*, 6567. https://doi.org/10.3390/ijms22126567.

7. Lan, Y., Ni, W., and Tai, G. (2022). Expression of MUC1 in different tumours and its clinical significance (Review). Mol. Clin. Oncol. *17*, 161. https://doi.org/10.3892/mco.2022.2594.

8. Wang, H., Borlongan, M., Hemminki, A., Basnet, S., Sah, N., Kaufman, H.L., Rabkin, S.D., and Saha, D. (2023). Viral vectors expressing interleukin 2 for cancer immunotherapy. Hum. Gene Ther. *34*, 878–895. https://doi.org/10.1089/HUM.2023.099.

9. Rosenberg, S.A. (2014). IL-2: The First Effective Immunotherapy for Human Cancer. J. Immunol. *192*, 5451–5458. https://doi.org/10.4049/jimmunol.1490019.

10. Goebeler, M.E., and Bargou, R.C. (2020). T cell-engaging therapies — BiTEs and beyond. Nat. Rev. Clin. Oncol. *17*, 418–434. https://doi.org/10.1038/s41571-020-0347-5.

11. Guo, Z.S., Lotze, M.T., Zhu, Z., Storkus, W.J., and Song, X.-T. (2020). Bi- and Tri-Specific T Cell Engager-Armed Oncolytic Viruses: Next-Generation Cancer Immunotherapy. Biomedicines *8*, 204. https://doi.org/10.3390/biomedicines8070204.

12. Renaud-Gabardos, E., Hantelys, F., Morfoisse, F., Chaufour, X., Garmy-Susini, B., and Prats, A.-C. (2015). Internal ribosome entry site-based vectors for combined gene therapy. World J. Exp. Med. *5*, 11–20. https://doi.org/10.5493/wjem.v5.i1.11.

13. Jiang, Y., Li, Y., and Zhu, B. (2015). T-cell exhaustion in the tumor microenvironment. Cell Death Dis. *6*, e1792. https://doi.org/10.1038/cddis.2015.162.

14. Singh, A., Dees, S., and Grewal, I.S. (2021). Overcoming the challenges associated with CD3+ T-cell redirection in cancer. Br. J. Cancer *124*, 1037–1048. https://doi.org/10.1038/s41416-020-01225-5.

15. Clubb, J.H.A., Kudling, T.V., Heiniö, C., Basnet, S., Pakola, S., Cervera Carrascón, V., Santos, J.M., Quixabeira, D.C.A., Havunen, R., Sorsa, S., et al. (2022). Adenovirus Encoding Tumor Necrosis Factor Alpha and Interleukin 2 Induces a Tertiary Lymphoid Structure Signature in Immune Checkpoint Inhibitor Refractory Head and Neck Cancer. Front. Immunol. *13*, 794251. https://doi.org/10.3389/fimmu.2022.794251.

16. Deng, J., Wang, L., Chen, H., Li, L., Ma, Y., Ni, J., and Li, Y. (2013). The role of tumour-associated MUC1 in epithelial ovarian cancer metastasis and progression. Cancer Metastasis Rev. *32*, 535–551. https://doi.org/10.1007/s10555-013-9423-y.

17. Ganesan, R., Chennupati, V., Ramachandran, B., Hansen, M.R., Singh, S., and Grewal, I.S. (2021). Selective recruitment of γδ T cells by a bispecific antibody for the treatment of acute myeloid leukemia. Leukemia *35*, 2274–2284. https://doi.org/10.1038/s41375-021-01122-7.

18. Foord, E., Arruda, L.C.M., Gaballa, A., Klynning, C., and Uhlin, M. (2021). Characterization of ascites- And tumor-infiltrating γδ T cells reveals distinct repertoires and a beneficial role in ovarian cancer. Sci. Transl. Med. *13*, eabb0192. https://doi.org/10.1126/SCITRANSLMED.ABB0192.

19. Kudling, T.V., Clubb, J.H.A., Quixabeira, D.C.A., Santos, J.M., Havunen, R., Kononov, A., Heiniö, C., Cervera-Carrascon, V., Pakola, S., Basnet, S., et al. (2022). Local delivery of interleukin 7 with an oncolytic adenovirus activates tumor-infiltrating lymphocytes and causes tumor regression. Oncoimmunology *11*, 2096572. https://doi.org/10.1080/2162402X.2022.2096572.

20. Havunen, R., Kalliokoski, R., Siurala, M., Sorsa, S., Santos, J.M., Cervera-carrascon, V., Anttila, M., and Hemminki, A. (2021). Cytokine-coding oncolytic adenovirus TILT-123 is safe, selective, and effective as a single agent and in combination with immune checkpoint inhibitor anti-PD-1. Cells *10*, 246. https://doi.org/10.3390/cells10020246.

21. Peterson, V.M., Castro, C.M., Chung, J., Miller, N.C., Ullal, A.V., Castano, M.D., Penson, R.T., Lee, H., Birrer, M.J., and Weissleder, R. (2013). Ascites analysis by a microfluidic chip allows tumor-cell profiling. Proc. Natl. Acad. Sci. USA *110*, E4978–E4986. https://doi.org/10.1073/PNAS.1315370110.

22. Scott, E.M., Frost, S., Khalique, H., Freedman, J.D., Seymour, L.W., and Lei-Rossmann, J. (2020). Use of Liquid Patient Ascites Fluids as a Preclinical Model for Oncolytic Virus Activity. Methods Mol. Biol. *2058*, 261–270. https://doi.org/10.1007/978-1-4939-9794-7_17.

23. Bains, S.J., and Yaqub, S. (2016). Characterization of Immunosuppressive Properties of Malignant Ascites in Ovarian Carcinoma. Gynecol. Obstet. *6*, 1–6. https://doi.org/10.4172/2161-0932.1000396.

24. Wefers, C., Duiveman-De Boer, T., Yigit, R., Zusterzeel, P.L.M., Van Altena, A.M., Massuger, L.F.A.G., and De Vries, I.J.M. (2019). Survival of ovarian cancer patients is independent of the presence of DC and T cell subsets in ascites. Front. Immunol. *10*, 3156. https://doi.org/10.3389/fimmu.2018.03156.

25. Kozłowski, M., Borzyszkowska, D., and Cymbaluk-Płoska, A. (2022). The Role of TIM-3 and LAG-3 in the Microenvironment and Immunotherapy of Ovarian Cancer. Biomedicines *10*, 2826. https://doi.org/10.3390/biomedicines10112826.

26. Mohamed, A.H., Obeid, R.A., Fadhil, A.A., Amir, A.A., Adhab, Z.H., Jabouri, E.A., Ahmad, I., and Alshahrani, M.Y. (2023). BTLA and HVEM: Emerging players in the tumor microenvironment and cancer progression. Cytokine *172*, 156412. https://doi.org/10.1016/j.cyto.2023.156412.

27. Ma, L., Sun, L., Zhao, K., Dong, Z., Huang, Z., and Meng, X. (2021). The prognostic value of TCF1+CD8+T in primary small cell carcinoma of the esophagus. Cancer Sci. *112*, 4968–4976. https://doi.org/10.1111/cas.15167.

28. Zhang, Z., Liu, S., Zhang, B., Qiao, L., Zhang, Y., and Zhang, Y. (2020). T Cell Dysfunction and Exhaustion in Cancer. Front. Cell Dev. Biol. *8*, 17. https://doi.org/10.3389/fcell.2020.00017.

29. Jhita, N., Raikar, S.S., and Program, G.T. (2022). Allogeneic gamma delta T cells as adoptive cellular therapy for hematologic malignancies. Explor. Immunol. *2*, 334–350. https://doi.org/10.37349/ei.2022.00054.

30. Li, Y., Zhou, C., Li, J., Liu, J., Lin, L., Li, L., Cao, D., Li, Q., and Wang, Z. (2018). Single domain based bispecific antibody, Muc1-Bi-1, and its humanized form, Muc1-Bi-2, induce potent cancer cell killing in muc1 positive tumor cells. PLoS One *13*, e0191024. https://doi.org/10.1371/journal.pone.0191024.

31. Zhao, Z., Zou, S., Guan, X., Wang, M., Jiang, Z., Liu, Z., Li, C., Lin, H., Liu, X., Yang, R., et al. (2018). Apolipoprotein E Overexpression Is Associated With Tumor Progression and Poor Survival in Colorectal Cancer. Front. Genet. *9*, 650. https://doi.org/10.3389/fgene.2018.00650.

32. Mack, D.L., Leibowitz, D.S., Cooper, S., Ramsey, H., Broxmeyerz, H.E., and Hromas, R. (2002). Down-regulation of the myeloid homeobox protein Hex is essential for normal T-cell development. Immunology *107*, 444–451.

33. Zhang, H., Sheng, L., Tao, J., Chen, R., Li, Y., Sun, Z., and Qian, W. (2016). Depletion of the triggering receptor expressed on myeloid cells 2 inhibits progression of renal cell carcinoma via regulating related protein expression and PTEN-PI3K/Akt pathway. Int. J. Oncol. *49*, 2498–2506. https://doi.org/10.3892/ijo.2016.3740.

34. Bulla, R., Tripodo, C., Rami, D., Ling, G.S., Agostinis, C., Guarnotta, C., Zorzet, S., Durigutto, P., Botto, M., and Tedesco, F. (2016). C1q acts in the tumour microenvironment as a cancer-promoting factor independently of complement activation. Nat. Commun. *7*, 10346. https://doi.org/10.1038/ncomms10346.

35. Foord, E., Arruda, L.C.M., Gaballa, A., Klynning, C., and Uhlin, M. (2021). Characterization of ascites-and tumor-infiltrating γδ T cells reveals distinct repertoires and a beneficial role in ovarian cancer. Sci. Transl. Med. *13*, 192.

36. Blanc, C., Hans, S., Tran, T., Granier, C., Saldman, A., Anson, M., Oudard, S., and Tartour, E. (2018). Targeting Resident Memory T Cells for Cancer Immunotherapy (Front. Media S.A.). https://doi.org/10.3389/fimmu.2018.01722.

37. Heiniö, C., Havunen, R., Santos, J., de Lint, K., Cervera-Carrascon, V., Kanerva, A., and Hemminki, A. (2020). TNFa and IL2 Encoding Oncolytic Adenovirus Activates Pathogen and Danger-Associated Immunological Signaling. Cells *9*, 798. https://doi.org/10.3390/cells9040798.

38. Zafar, S., Basnet, S., Launonen, I.-M., Carolina, D., Quixabeira, A., Santos, J., Hemminki, O., Malmstedt, M., Cervera-Carrascon, V., Aronen, P., et al. (2021). Oncolytic Adenovirus Type 3 Coding for CD40L Facilitates Dendritic Cell Therapy of Prostate Cancer in Humanized Mice and Patient Samples. Hum. Gene Ther. *32*, 192–202. https://doi.org/10.1089/hum.2020.222.

39. Kanerva, A., Zinn, K.R., Chaudhuri, T.R., Lam, J.T., Suzuki, K., Uil, T.G., Hakkarainen, T., Bauerschmitz, G.J., Wang, M., Liu, B., et al. (2003). Enhanced therapeutic efficacy for ovarian cancer with a serotype 3 receptor-targeted oncolytic adenovirus. Mol. Ther. *8*, 449–458. https://doi.org/10.1016/S1525-0016(03)00200-4.

YMTHE, Volume *32*

## Supplemental Information

# Overcoming effector T cell exhaustion in ovarian cancer ascites with a novel adenovirus encoding for a MUC1 bispecific antibody engager and IL-2 cytokine

**Saru Basnet, Mirte Van der Heijden, Dafne C.A. Quixabeira, Elise Jirovec, Susanna A.M. Grönberg-Vähä-Koskela, James H.A. Clubb, Anna Kanerva, Santeri Pakola, Lyna Haybout, Victor Arias, Otto Hemminki, Tatiana Kudling, Sadia Zafar, Victor Cervera-Carrascon, Joao M. Santos, and Akseli Hemminki**

**Table S1. List of antibodies used in this study.**

| Experiment | Antibodies | Manufacturer |
|---|---|---|
| T cell activation assay (in vitro) and immunological analysis of tumor (in vivo) | FITC anti-human CD3 (clone OKT3) | Biolegend, California, USA |
| | AF-700 anti- human CD4 (clone A161A1) | |
| | BV-421 anti- human CD8 (clone SK1) | |
| | PE-Cy™7 anti- human CD69 (clone FN50) | |
| | PE-Cy™5 anti- human CD25 (clone M-A251) | |
| | PE-CF594 anti-human CD127 (clone HIL-7R-M21) | BD Sciences, New Jersey, USA |
| | APC anti-human Tbet (clone 4B10) | Biolegend, California, USA |
| | PE anti-human EOMES (clone X4-83) | BD Sciences, New Jersey, USA |
| Expression of cell surface MUC1 antigen | APC anti-human MUC1 (clone 16A) | Biolegend, California, USA |
| Profiling of NK and NK-T like cells | BV-605 anti-human CD56 (HCD56) | Biolegend, California, USA |
| Activation of γδ T cell | PE anti-human TCR-γδ (clone 11F2) | Miltenyi, Bergisch Gladbach, Germany |
| | FITC anti-human Vδ1 (clone REA173) | |
| | APC anti-human Vδ2 (clone REA771) | |
| | AF-700 Perforin (clone B-D48) | |
| | BV421 GranzymeB (clone GB11) | BD Sciences, New Jersey, USA |
| | BV650 Interferon gamma (clone 4S.B3) | Biolegend, California, USA |
| T cell exhaustion panel | FITC anti-human CD3 (clone OKT3) | BioLegend, California, USA |
| | AF-700 anti-human CD4 (clone A161A1) | |
| | BV-510 anti-human CD8 (clone SK1) | BD Sciences, New Jersey, USA |
| | PE anti-human TIM-3 (clone A18087E) | BioLegend, California, USA |
| | BV-605 anti-human TIGIT (clone A15153G) | |
| | PE anti-human TIM-3 (clone A18087E) | |
| | BV-605 anti-human TIGIT (clone A15153G) | |
| | PE-Cy™5 anti-human LAG-3 (clone 11C3C65) | |
| | PE-Cy7 anti-human BTLA (clone MIH26) | |
| | APC anti-human CTLA-4 (clone BNI3) | |
| | PE-CF594 anti-human PD-1 (clone NAT105) | |
| | PE anti-human TOX (clone TXRX10) | Invitrogen, Massachusetts, USA |
| | BV-421 anti-human TCF-1 (clone S33-966) | BD Sciences, New Jersey, USA |

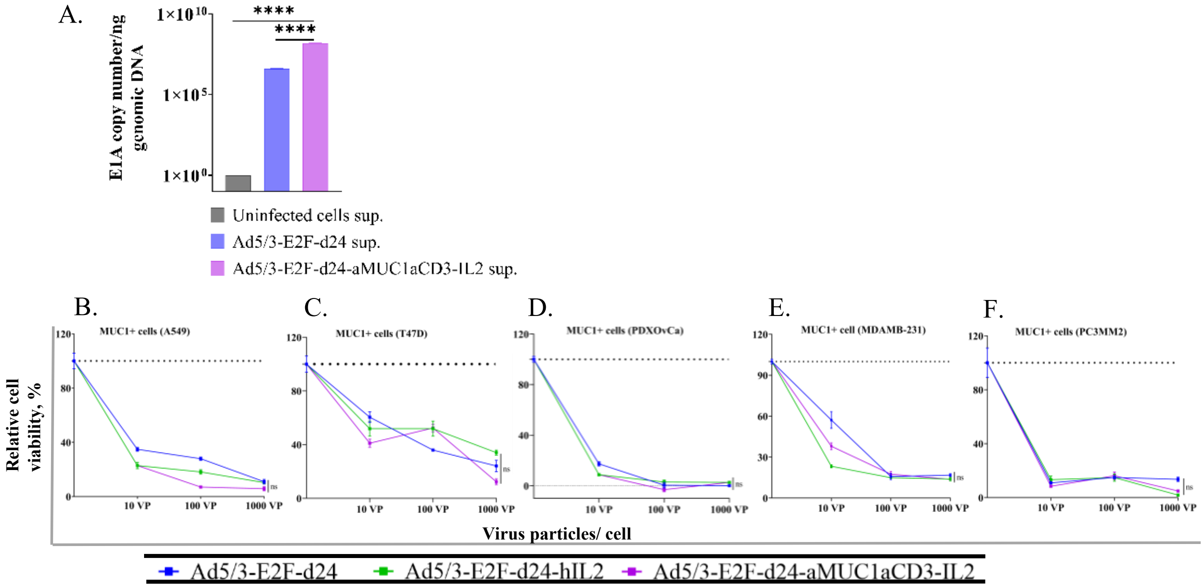


**Figure S1. Oncolytic activity of TILT-322 *in vitro*. (A)** Quantification of E1A gene copy number as a measure of virus replication in TILT-322 infected A549 cells supernatant. **(B-F)** Relative cell viability of human cancer cells followed by the infection of Ad5/3-E2F-d24, Ad5/3-E2F-d24-hIL2, and TILT-322 virus different concentration of virus ranging from 10, 100 or 1000 VP/ cell. Cells were incubated with the viruses for 3 days (A549, PDX-OvCa, and MDAMB-231), 5 days (PC3MM2), or 7 days (T47D) before determining cell viability. The untreated control group is indicated by the dashed line.

All experiments were run in quadruplet, and the resulting data are presented as mean ± SEM. One-way ANOVA with Tukey's post hoc test was used to compare more than two groups in the activation assay. Statistical significance is represented as *p < 0.05, ***p < 0.001, ****p < 0.0001 and ns = non-significant.

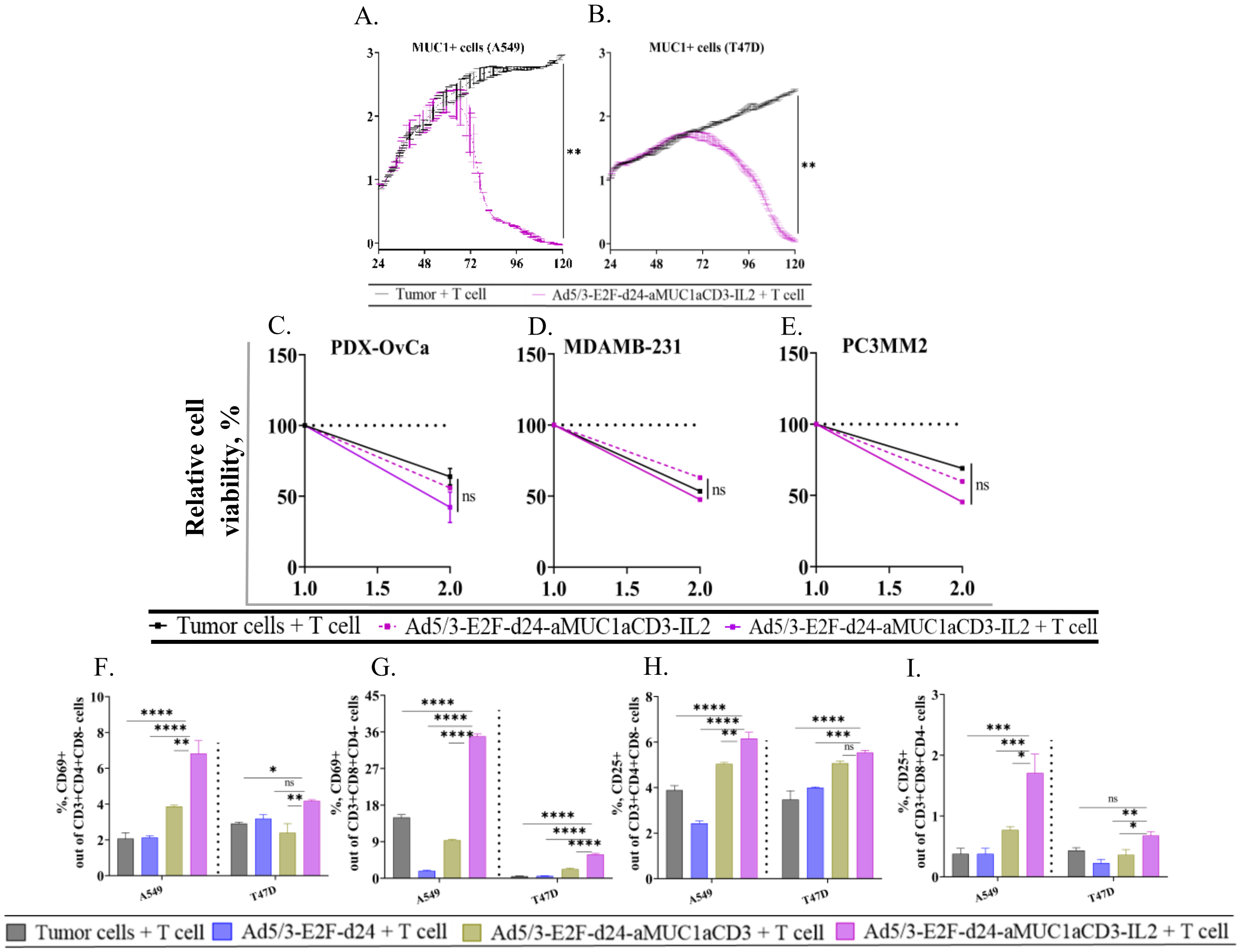
A.
MUC1+ cells (A549)
B.
MUC1+ cells (T47D)
Tumor + T cell
Ad5/3-E2F-d24-aMUC1aCD3-IL2 + T cell
C.
PDX-OvCa
D.
MDAMB-231
E.
PC3MM2
Relative cell viability, %
Tumor cells + T cell
Ad5/3-E2F-d24-aMUC1aCD3-IL2
Ad5/3-E2F-d24-aMUC1aCD3-IL2 + T cell
F.
%, CD69+ out of CD3+CD4+CD8- cells
G.
%, CD69+ out of CD3+CD8+CD4- cells
H.
%, CD25+ out of CD3+CD4+CD8- cells
I.
%, CD25+ out of CD3+CD8+CD4- cells
A549
T47D
Tumor cells + T cell
Ad5/3-E2F-d24 + T cell
Ad5/3-E2F-d24-aMUC1aCD3 + T cell
Ad5/3-E2F-d24-aMUC1aCD3-IL2 + T cell

**Figure S2**. **TILT-322 mediated tumor cell killing and T cell activation *in vitro*.** Real-time iCELLigence-based cytotoxicity assay showing the killing of MUC1-positive **(A)** A549 and **(B)** T47D tumor cells in the presence of virus and T cells at E:T ratio of 1 for up to 120 hours. **(C-E)** Evident of faster tumor cell killing by TILT-322 in the presence of unstimulated human CD3+ cells at E:T ratio of 5 and 1 pfu/cell virus concentration 48 hours post-infection. **(F-G)** Percentage of activated CD69+ and **(H-I)** CD25+ lymphocytes in TILT-322 treated tumors. All experiments were run in quadruplet, and the resulting data are presented as mean ± SEM. Welch's t-test was used to determine statistical significance in the iCELLigence assay and one-way ANOVA with Tukey's post hoc test was used to compare more than two groups in the activation assay. Statistical significance is represented as *p < 0.05, ***p < 0.001, ****p < 0.0001 and ns = non-significant. Statistical significance in xCELLigence assay is represented as *p = 0.0282, and **p = 0.0057.

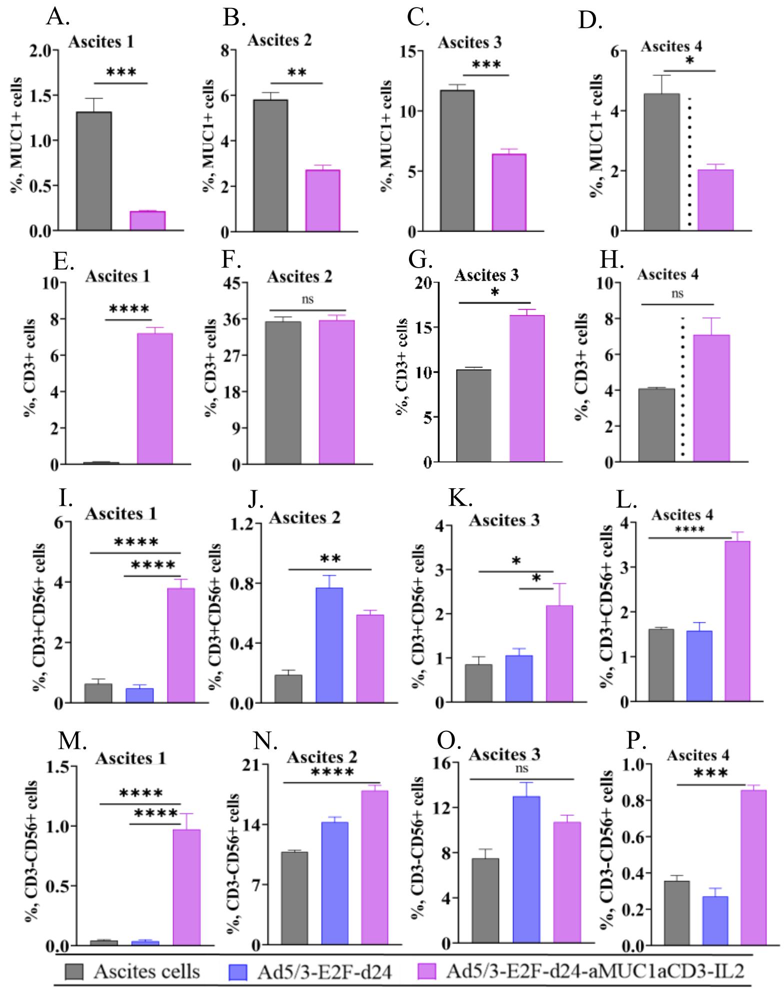


**Figure S3. Detection of MUC1, T cells, and other cells followed by TILT-322 infection in ascites samples. (A-D)** Detection of reduced proportion of MUC1 antigen in ascites cells and **(E-H)** Total percentage of CD3+ T cell detected in ascites samples **(I-L)** Total percentage of CD3+CD56+ cells and **(M-P)** CD3-CD56+ NK cells followed detected in four ascites samples infected with TILT-322 virus. The resulting data are presented as mean ± SEM of quadruplet. One-way ANOVA with Tukey's post hoc test was used to run the statistical significance and the result is represented as *$p < 0.05$, **$p < 0.01$, ***$p < 0.001$, and ****$p < 0.0001$.

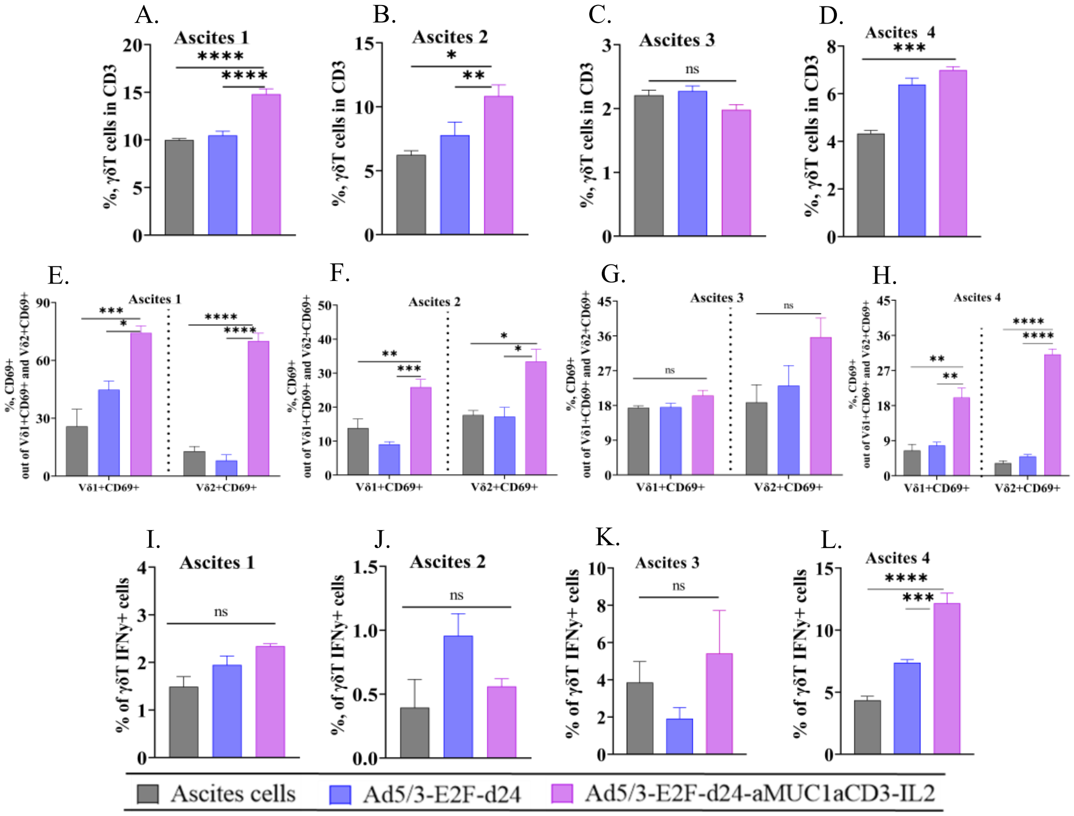


**Figure S4. TILT-322 mediated activation of γδT cell.**

The graph shows the percentage of **(A-D)** γδ T cells and **(E-H)** Percentage of activated CD69+ cells among the Vδ1+ and Vδ2+ subsets. **(I-L)** Interferon-gamma positive γδ T cells in all three tested ascites samples infected by TILT-322. The resulting data are presented as mean ± SEM of quadruplet. One-way ANOVA with Tukey's post hoc test was used to run the statistical significance is represented as ***$p < 0.001$, ****$p < 0.0001$ and, ns = non-significant.

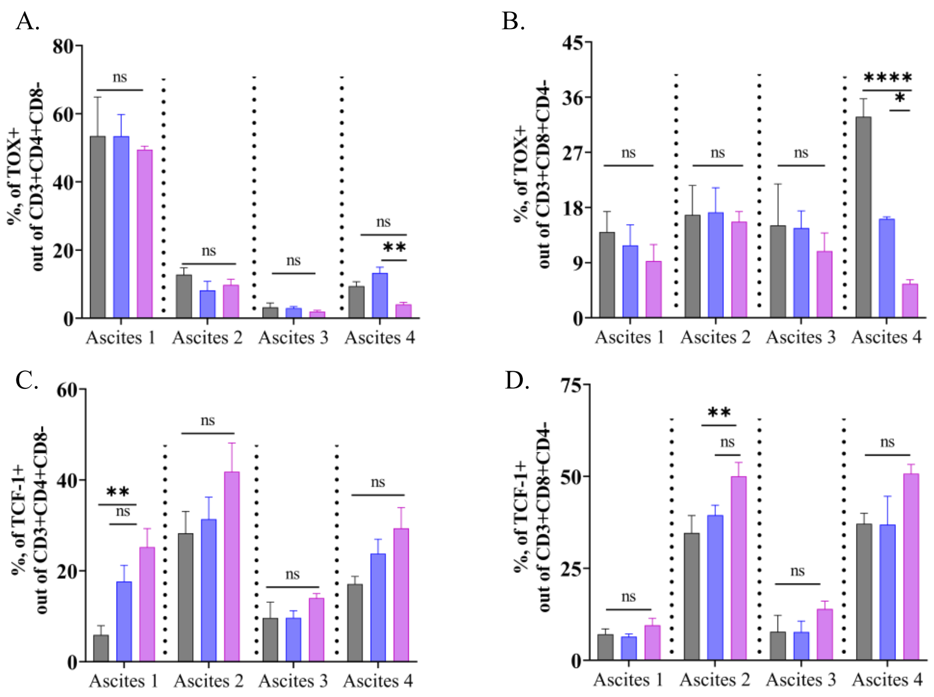


**Figure S5. T cell exhaustion at the transcriptional level followed by the TILT-322 treatment.** The graph shows the percentage of **(A-B)** TOX+ cells and **(C-D)** TCF-1+ cells on CD4+ and CD8+ T cells followed by TILT-322 treatment in four different ascites samples. The resulting data are presented as mean ± SEM of quadruplet. One-way ANOVA with Tukey's post hoc test was used to run the statistical significance is represented as ***p < 0.001, ****p < 0.0001 and, ns = non-significant.

A.

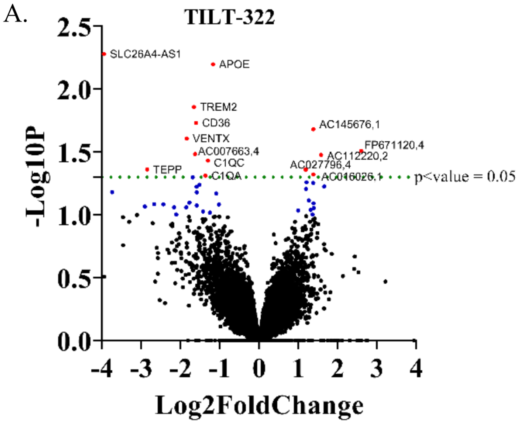


**Figure S6. Differentially expressed genes in TILT-322 infected ascites cells.** TILT-322 infection of ovarian ascites samples led to downregulation of 14 and upregulation of 5 differentially expressed genes in ascites samples.

A.

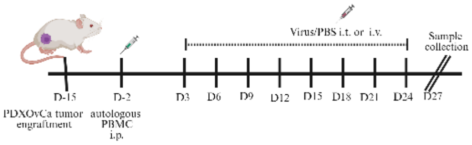


| Group | Dose | Route |
|---|---|---|
| PBMC | 1 x $10^7$ | Intraperitoneal |
| Ad5/3-E2F-d24 | 1 x $10^9$ | Intratumoral |
| Ad5/3-E2F-d24 aMUC1aCD3 | 1 x $10^9$ | Intratumoral |
| Ad5/3-E2F-d24 aMUC1aCD3-IL2 | 1 x $10^9$ | Intratumoral |
| Ad5/3-E2F-d24 aMUC1aCD3-IL2 | 1 x $10^{10}$ | Intravenous |

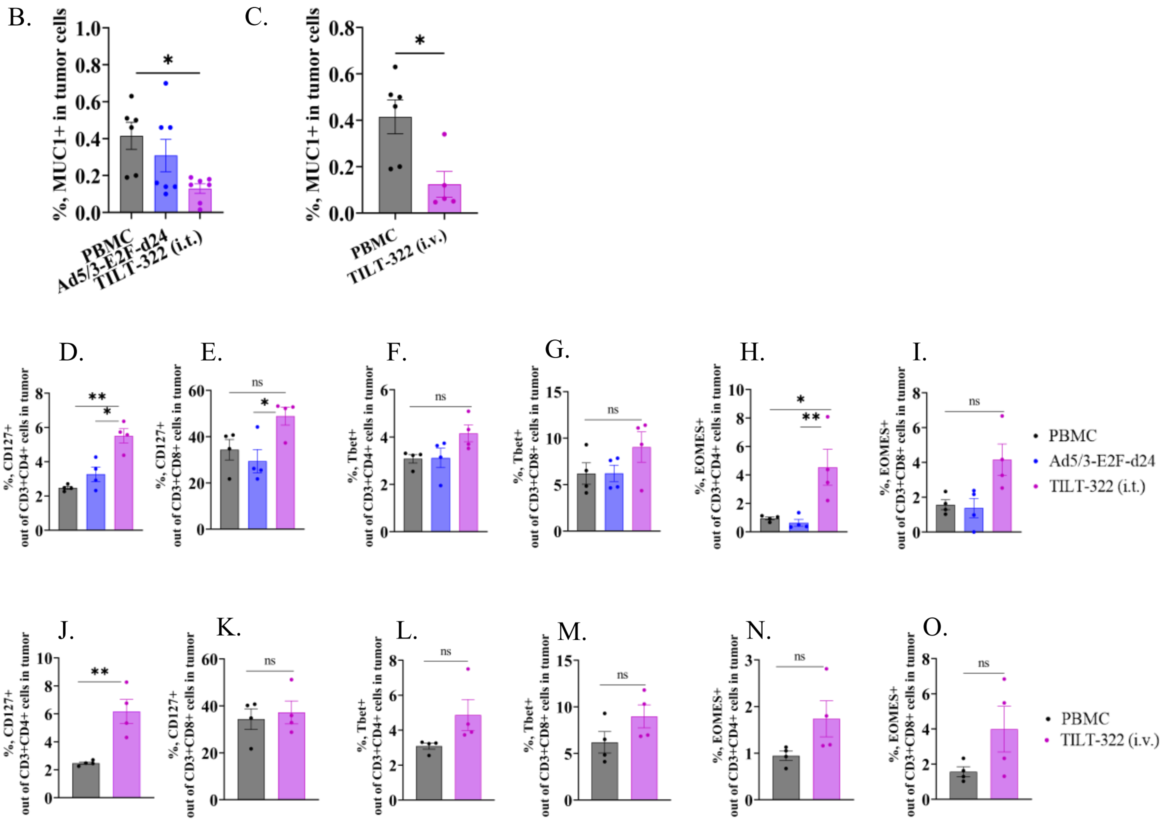

**Figure S7. Schedule for the treatment of TILT-322 *in vivo* experiment and immunological analysis of animal tumor samples. (A)** Schematic representation of TILT-322 animal experiment with dosage information. **(B)** Total percentage of MUC-1 positive cells detected in TILT-322 virus-treated tumors with intratumoral and **(C)** intravenous delivery. Total percentage of **(D-E)** CD127+ cells, **(F-G)** T-bet+ cells, **(H-I)** EOMES+ cells on CD4+ or CD8+ T cells in *in vivo* tumor samples followed by the local delivery of TILT-322. When the TILT-322 was given systemically, the total percentage of (**J-K)** CD127+ cells, **(L-M)** T-bet+ cells, **(N-O)** EOMES+ cells on CD4+ or CD8+ T cells in *in vivo* tumor samples is shown. The resulting data are presented as mean ± SEM of quadruplet. One-way ANOVA with Tukey's post hoc test was used to run the statistical significance is represented as ***$p < 0.001$, ****$p < 0.0001$, and, ns = non-significant.

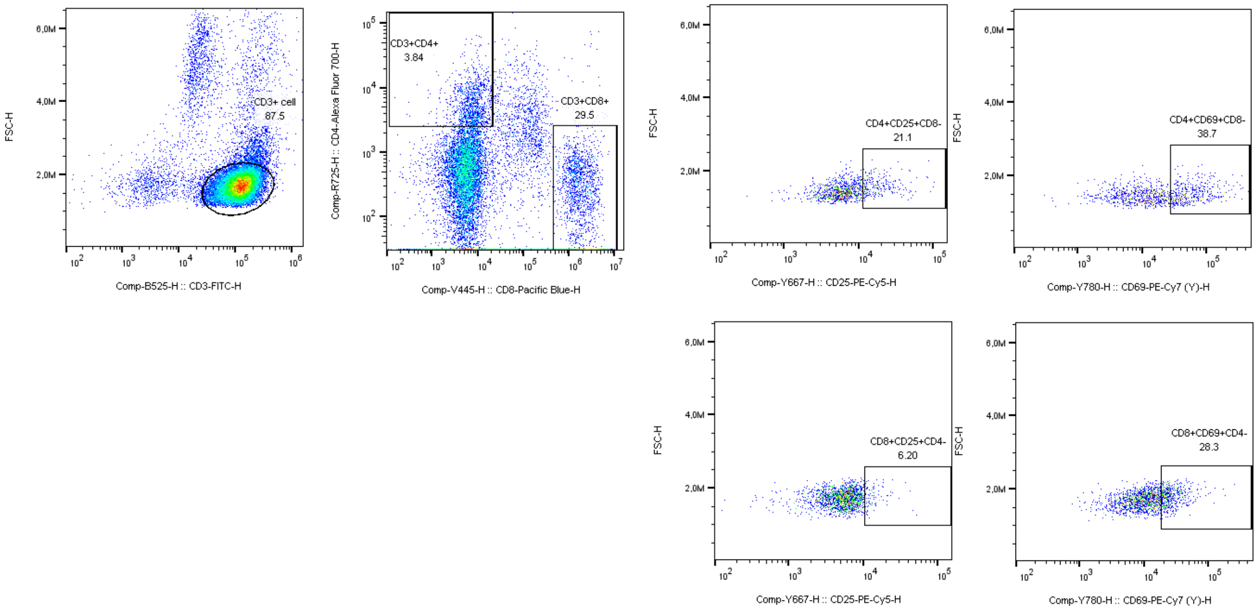


**Figure S8. Flow gating strategy used to access activated CD69 and CD25 T cells *in vitro*.** Gating is done from lymphocytes → CD3 → CD4 → CD69+/CD25+ or CD3 → CD8 → CD69+/CD25+.

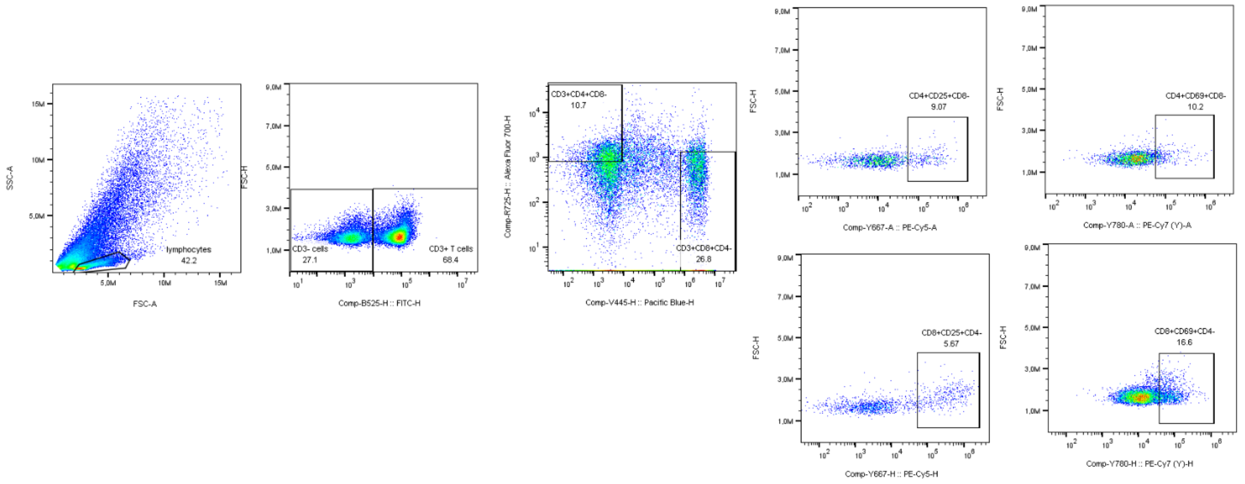


**Figure S9. Flow gating strategy used to access activated CD69 and CD25 T cell *ex vivo*.** Gating is done from lymphocytes→CD3+→CD4+→CD69+/CD25+ or →CD3+→CD8+→CD69+/CD25+.

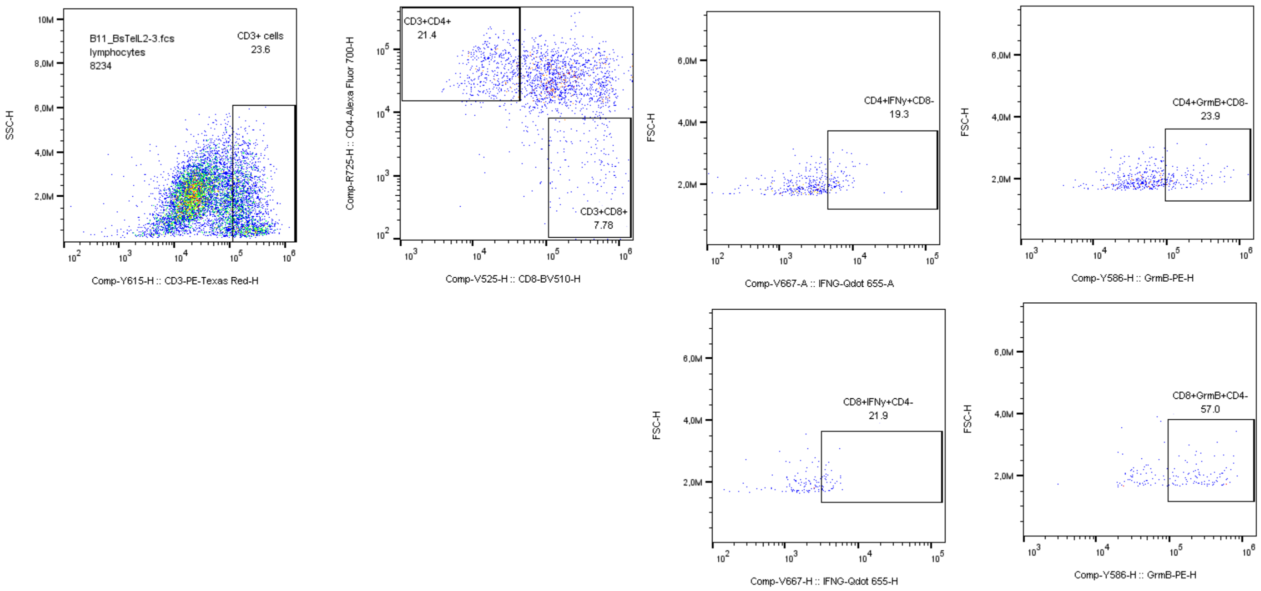


**Figure S10. Flow Gating Strategy used to access granzymeB and interferon-gamma positive cells in TILT-322 infected ascites samples.** Gating is done from lymphocytes→CD3+→CD4+→GranzymeB+/Interferon-y+ or →CD3+→CD8+→ GranzymeB+/Interferon-y+.

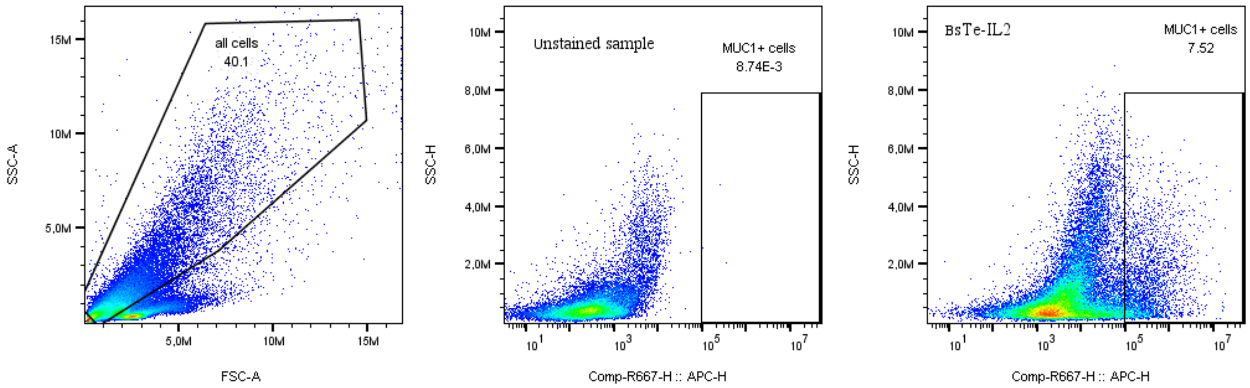


**Figure S11. Flow Gating Strategy used to access MUC1+ cells out of all ascites cells.** Gating is done from all cells → MUC1. MUC1 Fluorescence Minus One Control and unstained sample control were used to set the gating thresholds.

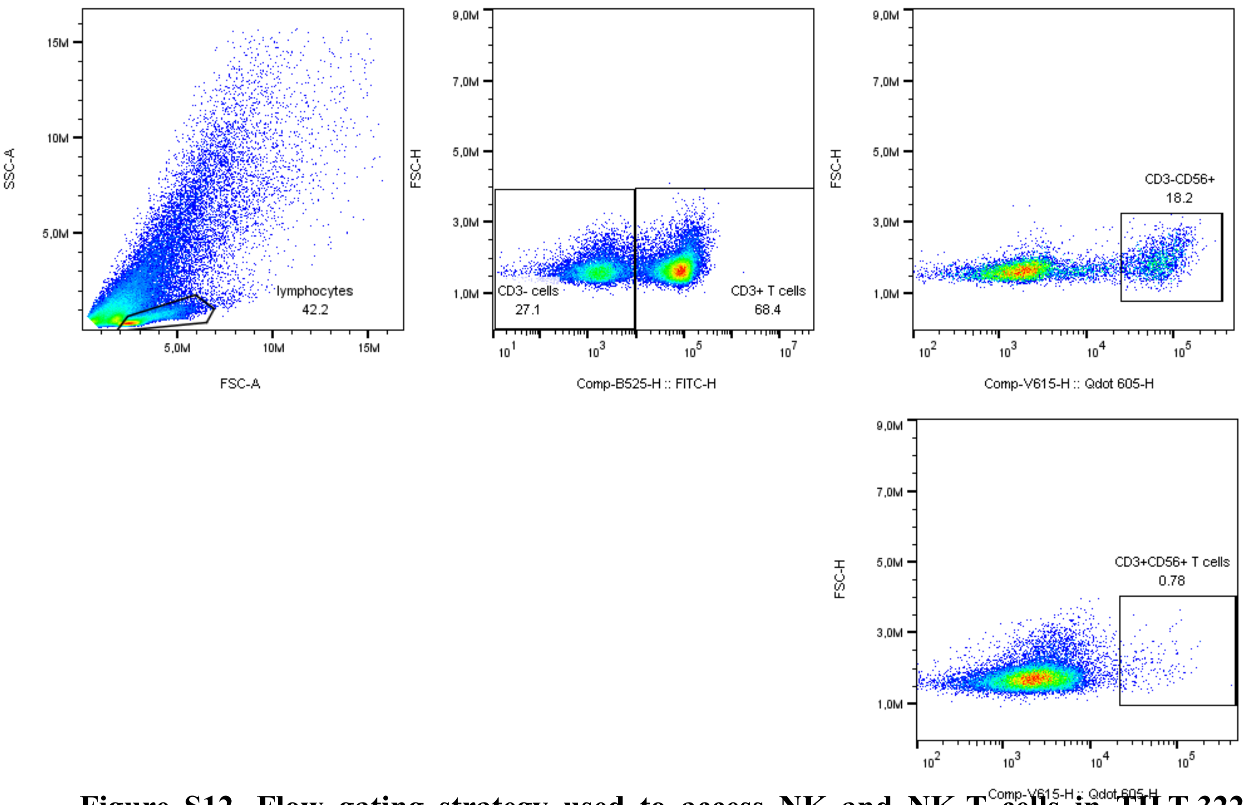


**Figure S12. Flow gating strategy used to access NK and NK-T cells in TILT-322 infected ascites samples.** Gating is done from lymphocytes → CD3+ cells → CD3+CD56+ cells or lymphocytes→ CD3- cells → CD3-CD56+ cells. All required Fluorescence Minus One Control and unstained sample control was used to set the gating thresholds.

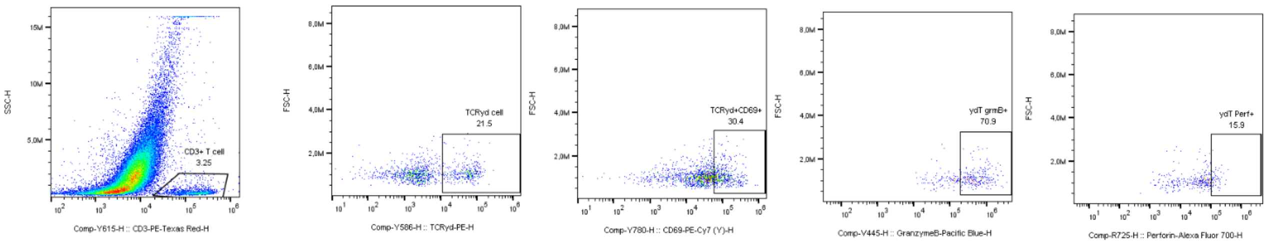


**Figure S13. Flow gating strategy used to access TCR gamma delta T cell activation in TILT-322 treated ascites samples *ex vivo*.** Gating is done from lymphocytes → CD3+ cells → TCRyd+ cells → CD69+ cells/ granzymeB+/ Perforin+ cells. All required Fluorescence Minus One Control and unstained sample control was used to set the gating thresholds.

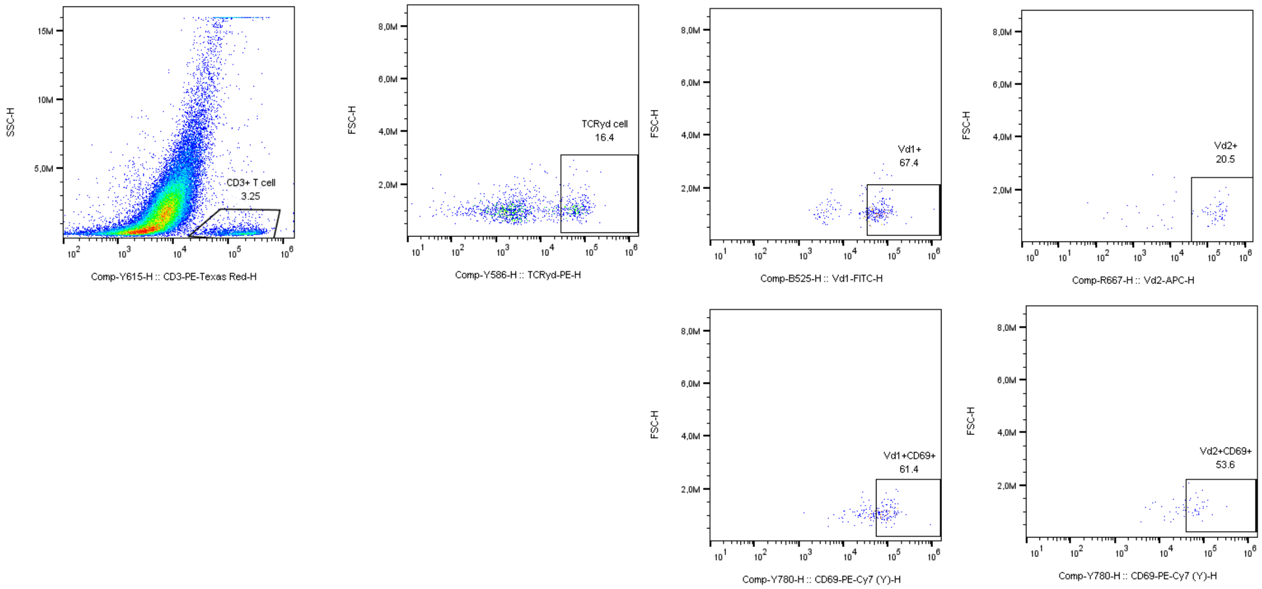


**Figure S14. Flow gating strategy used to access Vd1/Vd2 gamma delta T cell subset activation.** Gating is done from lymphocytes → CD3+ cells → TCRyd+ cells → Vd1+cells → CD69+ or lymphocytes → CD3+ cells → TCRyd+ cells → Vd2+cells → CD69+ cells. All required Fluorescence Minus One Control and unstained sample control was used to set the gating thresholds.

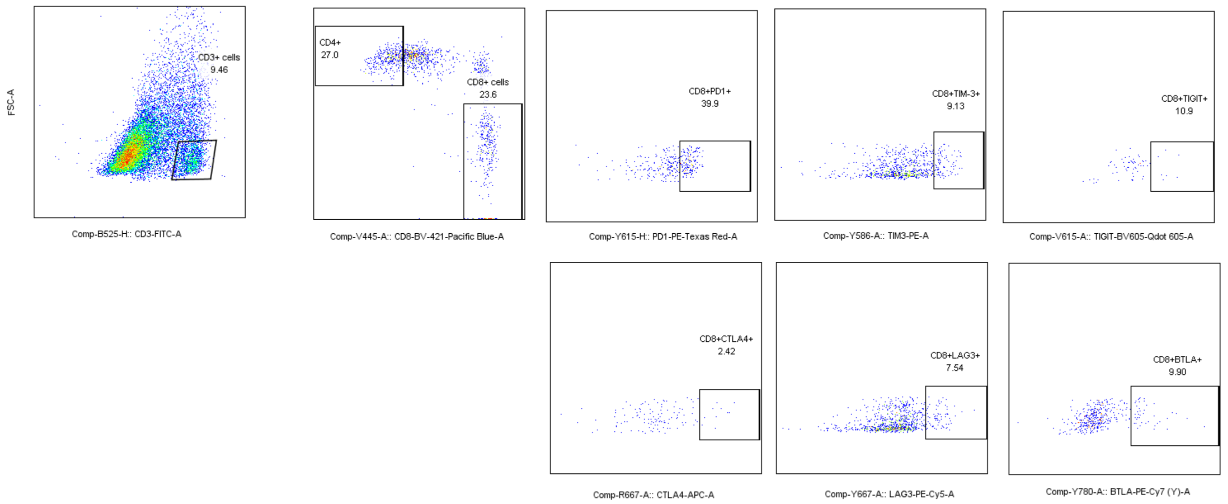


**Figure S15. Flow gating strategy used to examine T cell exhaustion in ovarian ascites followed by the TILT-322 treatment.** Gating is done from CD3+ cells → CD4+ → BTLA/CTLA-4/LAG-3/PD-1/TIGIT/TIM-3. A similar gating is done to access the expression on CD8+ T cells (CD3+ cells → CD8+ → BTLA/CTLA-4/LAG-3/PD-1/TIGIT/TIM-3). All required Fluorescence Minus One Control and unstained sample control was used to set the gating thresholds.
